# Evaluating Theoretical Solvent Models for Thermodynamic and Structural Descriptions of Dacarbazine–Cyclodextrin Complexes. The Theoretical and Conductometric Study

**DOI:** 10.3390/molecules30112309

**Published:** 2025-05-24

**Authors:** Zdzisław Kinart, Marta Hoelm, Martyna Imińska

**Affiliations:** Department of Physical Chemistry, Faculty of Chemistry, University of Lodz, Pomorska 163/165, 90-236 Lodz, Poland; martyna.iminska@edu.uni.lodz.pl

**Keywords:** cyclodextrins, drug delivery, enthalpy, Gibbs free energy of solvation, DFT calculations, solvent models, conductometry

## Abstract

In this study, the influence of various implicit solvent models on the structural and thermodynamic properties of dacarbazine complexes with three cyclodextrins—α-CD, HP-β-CD, and HE-β-CD—was evaluated. The models considered were the polarizable continuum model (PCM), the conductor-like polarizable continuum model (CPCM), the solvation model based on density (SMD), and the Onsager model. Theoretical thermodynamic results were compared with experimental data obtained from conductometric studies. Our findings indicated that all three cyclodextrins form stable 1:1 inclusion complexes with dacarbazine. Among them, the complexes with HE-β-CD were the most thermodynamically stable. While the choice of solvent model had a minor impact on the structural properties of the complexes, it significantly affected thermodynamic parameters such as enthalpy, Gibbs free energy, and solvation free energy. The best agreement with experimental data—particularly for the Gibbs free energy of solvation—was observed when using the SMD model.

## 1. Introduction

Understanding solvation effects is essential in accurately modeling chemical processes, particularly in solution-phase chemistry. One of the most widely employed approaches to account for solvent effects is the use of continuum solvation models [1], which treat the solvent not as discrete molecules but as a continuous polarizable medium characterized by macroscopic properties such as the dielectric constant. This simplification allows for the removal of explicit solvent molecules from simulations, provided that the continuum medium can replicate the key solvation properties [2,3]. Among the earliest and most foundational approaches is the Onsager reaction field model, which considers a solute placed in a spherical cavity within a uniform dielectric [4]. The solute’s permanent dipole induces a reaction field in the surrounding medium, which in turn interacts with the solute, stabilizing the system. This interaction is treated as a perturbation of the solute’s Hamiltonian and iteratively refined to reach self-consistency [4,5,6].

A more advanced class of continuum models includes polarizable continuum models (PCMs) [7], which extend the Onsager approach by incorporating not only electrostatic but also non-electrostatic interactions, such as dispersion, repulsion, and cavitation. The solvation free energy in PCM is commonly expressed as a sum of these individual contributions: [7,8,9]

(1) $$G=G_{cav}+G_{ele}+G_{dis}+G_{rep}+G_{thm}$$

where

G_cav_—is the free energy of cavity; G_ele_—is the free energy of electrostatic interactions; G_dis_—is the free energy of dispersion energy; G_rep_—is the free energy of repulsion energy and G_thm_—is the free energy of thermal fluctuations;

This decomposition allows for a more nuanced representation of solvation phenomena, particularly in polar solvents where electrostatic interactions dominate but other effects are still non-negligible [2,3,8]. PCMs have proven especially valuable in predicting properties like aqueous oxidation and reduction potentials, pKa values, and solvation energies in both aqueous and non-aqueous environments. Their ability to generate statistically averaged solvent effects within a single calculation makes them highly efficient and broadly applicable [3].

Several variants of PCM have been developed to enhance accuracy and flexibility. For instance, C-PCM implements a conductor-like approximation within the PCM framework [10], while SCIPCM determines the solute cavity from self-consistent isodensity surfaces [11,12].

A notable evolution of these models is the SMD (solvation model based on density), which incorporates the full quantum mechanical electron density of the solute rather than relying on partial atomic charges. The solvent is modeled as a dielectric continuum with a surface tension at the solute–solvent boundary. The SMD model partitions the solvation free energy into bulk electrostatic contributions and non-electrostatic terms, allowing it to be used universally across a wide range of solvents and solutes, both charged and neutral [13].

Despite their widespread use, relatively few comparative studies have systematically assessed the performance of implicit solvation models—particularly for computing aqueous oxidation potentials of diverse organic compounds—though such evaluations are common for solvation energies of closed-shell species [3]. This gap highlights the need for further investigation into the reliability and predictive capabilities of different implicit models in this context.

**Figure 1:**
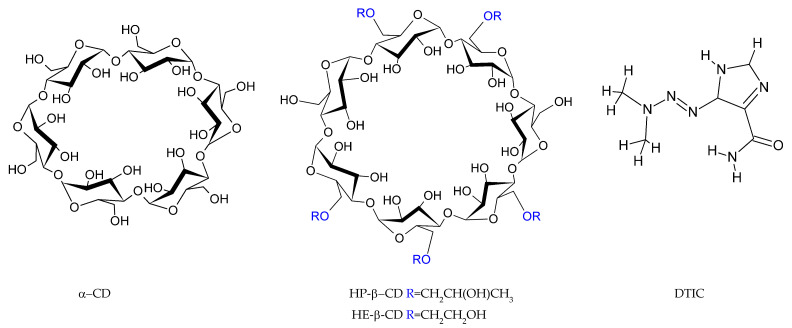
The schematic representation of α-CD, HP-β-CD, HE-β-CD, and dacarbazine (DTIC).

In our work, we performed comparison of implicit and explicit (water molecules were added into the investigated system) solvent models on the structural and thermodynamical properties of dacarbazine (DTIC) complex with three cyclodextrins: α-CD; HP-β-CD and HE-β-CD (Figure 1). Dacarbazine is an FDA-approved [14,15] alkylating chemotherapeutic agent primarily used in the treatment of metastatic melanoma and Hodgkin lymphoma [16,17]. It acts by disrupting cancer cell DNA, leading to cell death [18]. Despite its clinical relevance, DTIC use is limited by several drawbacks, including poor water solubility, low absorption, and non-selective cytotoxicity, which can result in side effects such as myelosuppression and gastrointestinal toxicity [17,19].

Its therapeutic response in melanoma remains low (10–20%), and treatment often requires high doses due to the acidic tumor environment, increasing the risk of drug resistance [16,18]. Consequently, DTIC is now mainly used in combination regimens, such as ABVD, or in patients unresponsive to targeted therapies [19,20,21,22,23,24]. Nevertheless, cytotoxicity of such regimens is also observed [25]. Novel strategies, including nanocarrier-based delivery and molecular adjuvants like miRNA, are being explored to enhance DTIC efficacy and selectivity [25,26,27,28,29].

Cyclodextrin inclusion complexes are widely used in various fields, including medicine, pharmacy, and materials science. For example, Moyers-Montoya and colleagues (2021) investigated the use of polycaprolactone nanofibers with an anchored epithelial growth factor on 6-deoxy-6-amino-β-cyclodextrin, demonstrating their effectiveness in both in vitro and in vivo studies [30]. Additionally, Escobedo-González et al. (2023) conducted in silico studies on novel cyclodextrin inclusion complexes with polycaprolactone, showing their potential in skin regeneration [31]. These studies highlight the importance of inclusion complexes in enhancing therapeutic and material properties.

Cyclodextrin inclusion complexes play a key role in improving the bioavailability and stability of drugs, which is especially important for compounds with low water solubility, such as dacarbazine. Cyclodextrins can form inclusion complexes with various molecules, enabling protection against degradation, enhanced solubility, and controlled release within the body. In the study by Moyers-Montoya et al. [30], polycaprolactone nanofibers with an anchored epithelial growth factor on 6-deoxy-6-amino-β-cyclodextrin were shown to be effective in tissue regeneration, confirmed by in vitro and in vivo experiments. Similarly, Escobedo-González et al. (2023) performed in silico analyses indicating that new cyclodextrin inclusion complexes with polycaprolactone have promising potential for skin regeneration, which may be crucial in wound and burn therapy [31].

In the context of our research, dacarbazine-cyclodextrin inclusion complexes may significantly improve their pharmacokinetic and pharmacodynamic properties. Dacarbazine, as an anticancer drug, exhibits poor water solubility and limited bioavailability, preventing its effective clinical use. The formation of inclusion complexes with cyclodextrins can enhance its solubility and stability and allow controlled release, potentially improving therapeutic efficacy and reducing adverse effects. Cyclodextrin inclusion complexes have broad applications in various fields, and their role in enhancing therapeutic and material properties is invaluable. Our research on dacarbazine-cyclodextrin complexes fits this context, aiming to improve the effectiveness and safety of anticancer therapies.

## 2. Results and Discussion

### 2.1. Analysis of Structural Properties of Dacarbazine-Theoretical Investigation

Although the structure of dacarbazine is available in the Cambridge Structural Database (CSD), we performed an extensive conformational search to identify the most stable conformer. This approach was motivated by our recent study [32], in which we investigated another anticancer drug, carmustine. There, we demonstrated that the conformer obtained through conformational analysis was more stable than the one retrieved from CSD. Consequently, a detailed conformational analysis was conducted for DTIC using a range of computational methods with progressively increasing levels of theoretical theory.

The initial step involved molecular mechanics calculations using the AMBER99 force field, which facilitated the identification of a set of energetically favorable conformers on the potential energy surface. In the second step, we employed semiempirical methods—PM6-D3-H4 and PM7 (see Materials and Methods)—commonly used for studying organic compounds. Notably, PM6-D3-H4 includes additional corrections for dispersion and hydrogen-bond interactions. Calculations were performed in water described by the conductor-like screening model (COSMO).

The geometries obtained from the semiempirical methods were further refined using the density functional theory (DFT) method. Several DFT methods were tested, including M08-HX-D3/aug-cc-pVTZ, M08-HX-D3/6-31G(d,p), DSD-PBEP86-D3BJ/aug-cc-pVTZ, and B97-D3/aug-cc-pVTZ. The 6-31G(d,p) basis set was specifically included because it was used in studies involving DTIC–cyclodextrin complexes (the Dunning basis set is too large to use in analysis of such systems). All DFT calculations were performed in water, with solvation effects modeled using the polarizable continuum model (PCM).

**Figure 2:**
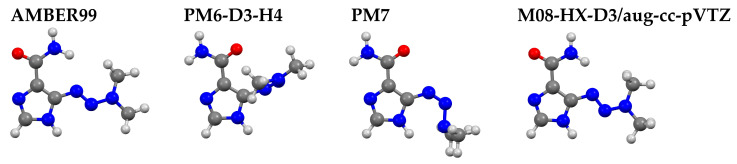
Conformers of DTIC identified using molecular mechanic (AMBER99), semiempirical methods (PM7 and PM6-D3-H4), and the DFT method (M08-HX-D3/aug-cc-pVTZ). Calculations were carried out in vacuo (AMBER99) and in water, described using the COSMO model (for the semiempirical level) and the PCM model (for the DFT level). Atom color scheme: carbon—dark gray, nitrogen—blue, oxygen—red, hydrogen—light gray.

The most stable structures obtained from each level of theory—molecular mechanics, semiempirical, and DFT—are shown in Figure 2. Interestingly, the conformer predicted by the AMBER99 force field showed better agreement with the DFT results than those obtained via semiempirical methods, despite their higher theoretical level. Furthermore, PM6-D3-H4 and PM7 pointed to different conformers as the most stable. In the geometry of the DTIC-1 conformer predicted by M08-HX-D3, the orientation of the carboxamide group toward the 3,3-dimethyltriaz-1-en-1-yl moiety enabled the formation of a moderate N–H···N hydrogen bond, as classified by the Jeffrey categorization based on H···A bond distance [33].

**Figure 3:**
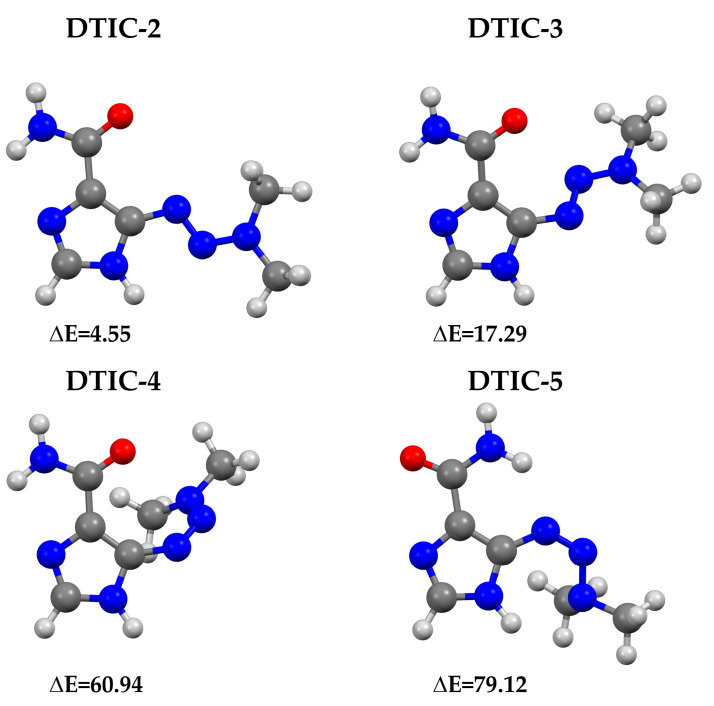
Less stable conformers of dacarbazine (DTIC) obtained at the M08-HX-D3/aug-cc-pVTZ theory level in water (PCM). The relative energies (ΔE, in kJ/mol) were calculated with respect to the most stable conformer, DTIC-1, presented in Figure 2.

Figure 3 presents other, less stable conformers of DTIC obtained at the M08-HX-D3/aug-cc-pVTZ level of theory. Interestingly, the trend observed in DTIC-1, where the carboxamide group was oriented toward the 3,3-dimethyltriaz-1-en-1-yl moiety, was not dominant across these structures. In fact, the first three less stable conformers (DTIC-2, DTIC-3, and DTIC-4) exhibited geometries more similar to those predicted by semiempirical methods (see Figure 2). The privileged geometry of DTIC-1 may result from the rotational barriers of the carboxamide and 3,3-dimethyltriaz-1-en-1-yl groups. These rotational barriers were investigated at the M08-HX-D3/aug-cc-pVTZ theoretical level within the PCM model. The analysis was performed around the C–C bond for the carboxamide group and around the C–N bond for the 3,3-dimethyltriaz-1-en-1-yl group (see Figure S1). The barriers were examined over a range of 0° to 360°, with a step size of 10°. Figure S1 also presents the rotational barriers (ΔE_rot_), calculated as the energy difference between the most and least stable conformations.

For the carboxamide group, the most stable conformation was observed at 0°, while the least stable occurred at 90°. In the case of the 3,3-dimethyltriaz-1-en-1-yl group, the energy minimum was reached at 180°. In DTIC-1 (obtained at M08-HX-D3; Figure 2), the carboxamide group adopted an angle of 0°, while the 3,3-dimethyltriaz-1-en-1-yl group adopted an angle of 180°.

In Figure S2 are displayed the relative energy differences (ΔE) calculated with respect to DTIC-1, whose geometry is shown in Figure 2 (indicated by DFT). The consistent exponential increase in energy across all methods indicates that the conformer ranking remained unchanged, further confirming the reliability of the M08-HX-D3/aug-cc-pVTZ results.

It is worth comparing our findings with those reported in the literature. In some studies [17,34,35], DTIC-2 (see Figure 3) is identified as the most stable conformer. However, in [36], both DTIC-1 and DTIC-2 are presented, with DTIC-1 noted as being more stable. The discrepancy suggesting DTIC-2 is energetically preferred may stem from the absence of a comprehensive conformational search in those studies—optimizations were based on the experimental geometry alone. Although the energy difference between DTIC-1 and DTIC-2 is relatively small (ΔE_M08-HX-D3/aug-cc-pVTZ_ = 4.55 kJ/mol; see Figure 3), it has a significant impact on the subsequent evaluation of DTIC complex stability. Therefore, in our study, the DTIC-1 structure was selected as the starting geometry for building complexes with α-CD, HP-β-CD, and HE-β-CD.

The DTIC-1 conformer presented in Figure 2 was obtained through DFT calculations in the presence of water, modeled using the implicit PCM solvent model. We also investigated whether employing an explicit solvent model would significantly affect the geometry of DTIC. Figure S3 shows dacarbazine surrounded by 20 water molecules DTIC-1 (20 H_2_O), for which we calculated the root mean square deviation (RMSD) of atomic positions relative to DTIC-1 (PCM). The RMSD value of 1.729 Å indicates that the explicit solvent model had a noticeable influence on molecular geometry. This effect was likely due to the formation of numerous hydrogen bonds between water molecules and DTIC-1, which alters the final geometry. The most prominent difference between DTIC-1 (PCM) and DTIC-1 (20 H_2_O) was observed in the 3,3-dimethyltriaz-1-en-1-yl group, which was rotated by approximately 45° in DTIC-1 (20 H_2_O).

### 2.2. The Influence of Solvent Models on the Complexation Stability of Cyclodextrin Complexes—Theoretical Study

**Figure 4:**
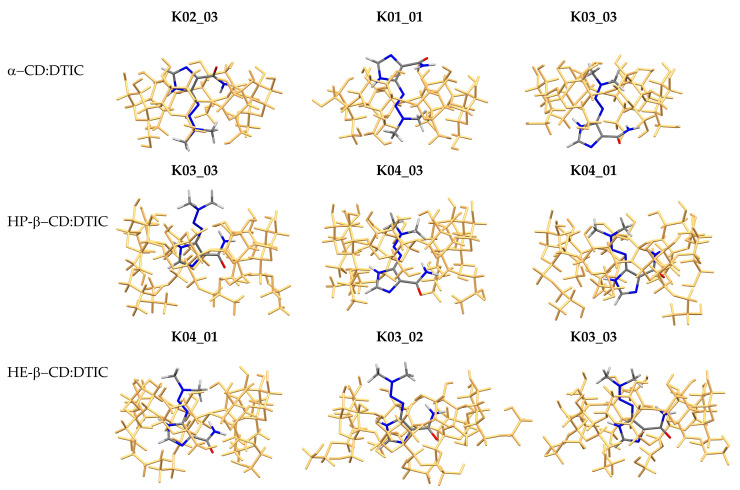
The most stable complexes of α-CD:DTIC, HP-β-CD:DTIC, and HE-β-CD:DTIC obtained at the M08-HX-D3/6-31G(d,p) theory level using the PCM solvent model. Cyclodextrins are highlighted in dark yellow. The total energy values and coordinates are listed in Tables S1 and S2, respectively.

In our study, we investigated the stability of DTIC complexes with various cyclodextrins: α-CD, HP-β-CD, and HE-β-CD. Additionally, we examined the influence of different implicit solvent models (PCM, CPCM, SMD, and Onsager) on the geometry of these complexes. The three most stable complexes for each type of cyclodextrin are presented in Figure 4. The notation used in this article for the complexes is K0X_0Y, where **X** ranges from 1 to 4 and **Y** from 1 to 3. K0X refers to the orientations K01, K02, K03, and K04 (see Figure S4), while 0Y denotes the specific structure—01, 02, or 03—analyzed within each orientation.

All structures shown in Figure 4 were obtained using the PCM model. Structures optimized with the other solvent models are not included, as no significant geometric differences were observed compared to PCM. This conclusion is supported by RMSD values calculated relative to the PCM-optimized geometries, which are listed in Table 1. As shown, the RMSD values were nearly zero, confirming the minimal impact of solvent model choice on the final geometry.

From each complex (α-CD:DTIC, HP-β-CD:DTIC, and HE-β-CD:DTIC), we selected the most stable structure—K02_03 (α-CD:DTIC), K03_03 (HP-β-CD:DTIC), and K04_01 (HE-β-CD:DTIC)—and performed calculations using an explicit solvent model. The detailed computational procedure is described in the Materials and Methods Section. Each complex was solvated with 20 water molecules.

Unfortunately, we were only able to obtain results for the α-CD:DTIC complex. For the remaining complexes, convergence issues occurred, most likely due to the larger size of the systems and the number of water molecules involved. The α-CD:DTIC complex with explicitly added water molecules is shown in Figure S5. The geometry of the complex did not change significantly upon solvation. Most structural changes were observed in the α-CD moiety rather than in the DTIC molecule.

**Figure 5:**
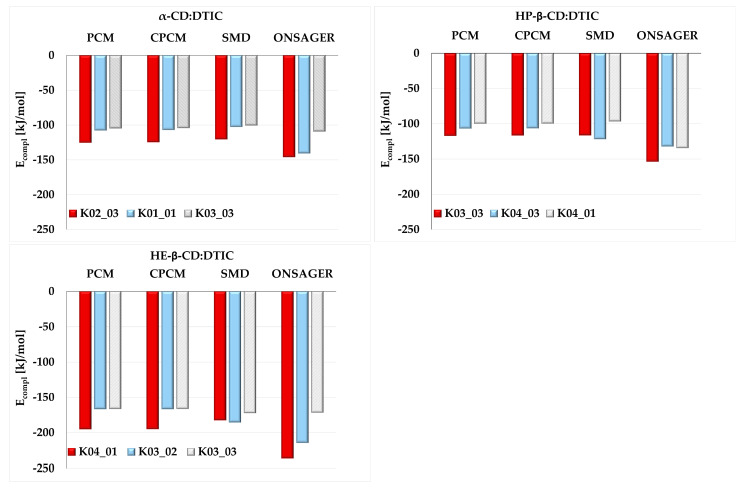
Complexation energies (E_compl_) [kJ/mol] obtained at the M08-HX-D3/6-31G(d,p) theory level using different solvent models (PCM, CPCM, SMD, and Onsager) for the three most stable complexes of α-CD:DTIC, HP-β-CD:DTIC, and HE-β-CD:DTIC.

As shown in Figure 4, our study examined various orientations of DTIC within the cyclodextrin (CD) cavities (see also Figure S4). In K01 and K02, the imidazole ring of DTIC was positioned near the wider rim of the CD cone, whereas in K03 and K04 it was oriented towards the narrower rim. Among the analyzed orientations, K03 and K04 were the most frequently observed. An exception was α-CD, for which two distinct orientations—K01 and K02—are presented. Both HP-β-CD:DTIC and HE-β-CD:DTIC possess additional substituent chains (hydroxypropyl and hydroxyethyl, respectively), which enable further interactions with DTIC. These interactions may positively affect the stability of the complexes. For example, HE-β-CD formed the most stable complex with DTIC, with a complexation energy (E_compl_) of approximately −194 kJ/mol (K04_01, PCM model; see Figure 5) or −236 kJ/mol (K04_01, Onsager model; see Figure 5). Interestingly, despite being the smallest among the studied CDs, α-CD formed highly stable complexes with DTIC, with stability comparable to that of the largest CD studied, HP-β-CD.

The influence of solvent models on the stability ranking of configurations was minimal. For the α-CD:DTIC complex, all models agreed that the K02_03 complex was the most stable. However, this consensus was not observed for the HP-β-CD:DTIC and HE-β-CD:DTIC complexes. While PCM, CPCM, and Onsager models pointed to the same complex, SMD identifies a different one; nevertheless, the ΔE_compl_ was relatively small. For instance, in the case of the HP-β-CD:DTIC complex, PCM indicated that K03_03 was the most stable, while SMD pointed to K04_03. The ΔE_compl_ calculated between these two structures was 5.5 kJ/mol.

In the final aspect of the geometry analysis, it was interesting to examine the formation of hydrogen bonds. Cyclodextrins form numerous intramolecular hydrogen bonds between sugar units. According to calculations, the presence of DTIC does not significantly affect the intramolecular hydrogen bonds present in cyclodextrins. However, their geometric parameters changed, albeit not greatly, as a result of the deformation of cyclodextrins occurring during the formation of the complex. For the most stable complexes selected from the PCM calculations—α-CD:DTIC K02_03, HP-β-CD:DTIC K03_03, and HE-β-CD:DTIC K04_01—we calculated the deformation energy (E_def_) as the energy difference between the monomer’s structure within the complex and its optimized isolated configuration. The deformation energy equaled 29.47 kJ/mol, 28.28 kJ/mol, and 0.9505 kJ/mol for α-CD:DTIC K02_03, HP-β-CD:DTIC K03_03, and HE-β-CD:DTIC K04_01, respectively. This indicates that the strongest deformation was observed in α-CD and HP-β-CD. In these cyclodextrins, nine hydrogen bonds (HB) were observed between DTIC and CDs. Conversely, in HE-β-CD:DTIC K04_01, twelve hydrogen bonds were formed, which positively affected the stability of the complex to some extent, as HE-β-CD formed the most stable complex with DTIC.

### 2.3. The Influnce of Solvent Models on the Thermodynamic Properties of Cyclodextrin Complexes-Comparison Between Theoretical and Experimental Study

**Figure 6:**
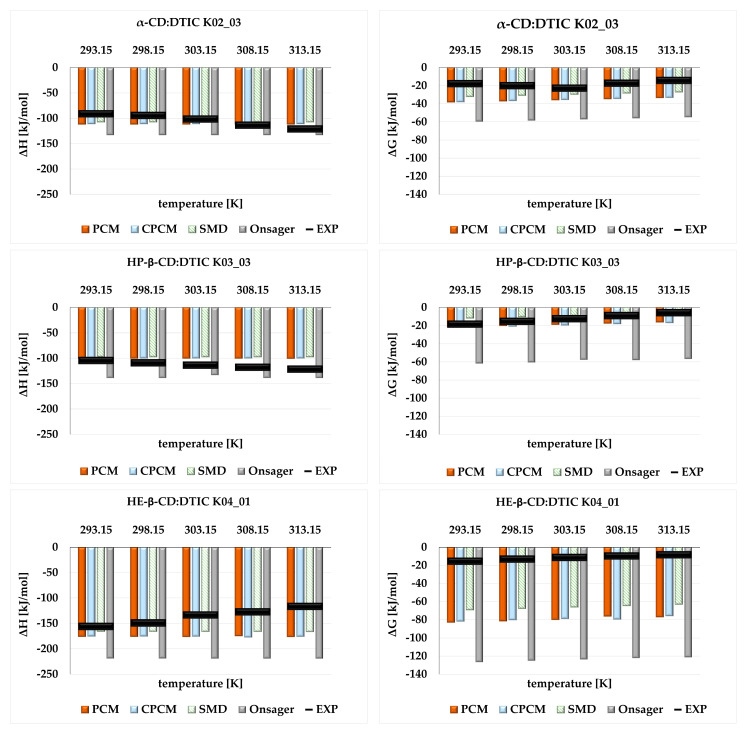
ΔH and ΔG [kJ/mol] calculated for the most stable complexes α-CD:DTIC K02_03, HP-β-CD:DTIC K03_03, and HE-β-CD:DTIC K04_01, obtained using different theoretical solvent models (PCM, CPCM, SMD, and Onsager). Experimental values (EXP) are marked with a black line. The analysis was performed in the temperature range 293.15–313.15 K.

In the final part of our study, we investigated how different theoretical solvent models influence thermodynamic properties such as complexation enthalpy (ΔH), Gibbs free energy (ΔG), and Gibbs free energy of solvation (ΔG_solv_). A comparison between theoretical and experimental values is presented in Figure 6 and Table 2, Table 3 and Table 4. The analysis was performed in the temperature range of 293.15–313.15 K.

Figure 6 compares the theoretical values obtained for the most stable structure of each complex with experimental data, while Table 2, Table 3 and Table 4 show values for two other complexes. In general, both the computational and conductometric measurements indicated that the formation of all complexes was exothermic and spontaneous, as reflected by the negative values of ΔH and ΔG, respectively. According to the experimental data, with increasing temperature, larger changes in ΔH and ΔG (i.e., the reaction becoming more or less exothermic or spontaneous) were observed than those predicted by the theoretical model.

Although the convergence between theoretical and experimental values was generally acceptable, some discrepancies were observed. These are especially noticeable in Figure 6, which presents values for only the most stable complexes in each set. Regardless of the parameters considered (ΔH and ΔG), the Onsager model significantly overestimated the thermodynamic data, particularly ΔG. CPCM and PCM are very similar solvent models (in terms of parametrizations), and their values did not differ significantly. However, when compared to SMD, it can be concluded that the latter model showed slightly better agreement with the experimental data.

The largest discrepancies between experimental and theoretical data were seen for ΔG estimated for HE-β-CD:DTIC K04_01. This can be explained by the coexistence of multiple molecular configurations in real solutions, including both stable and metastable forms. Hence, the experimental observations represent a superposition of their respective effects. This is particularly evident in Table 2, Table 3 and Table 4, where theoretical data for two other stable structures are shown. The convergence between calculated and measured values was better than for the most stable structures. For instance, the ΔG estimated for α-CD:DTIC K01_01 is −17.98 kJ/mol (293.15 K), while the experimental value was −17.85 kJ/mol. A similar trend was observed for HE-β-CD:DTIC, where for the complex ranking fourth in stability (K04_02), the ΔG was −15.46 kJ/mol (293.15 K), and the experimental value was −15.49 kJ/mol.

**Figure 7:**
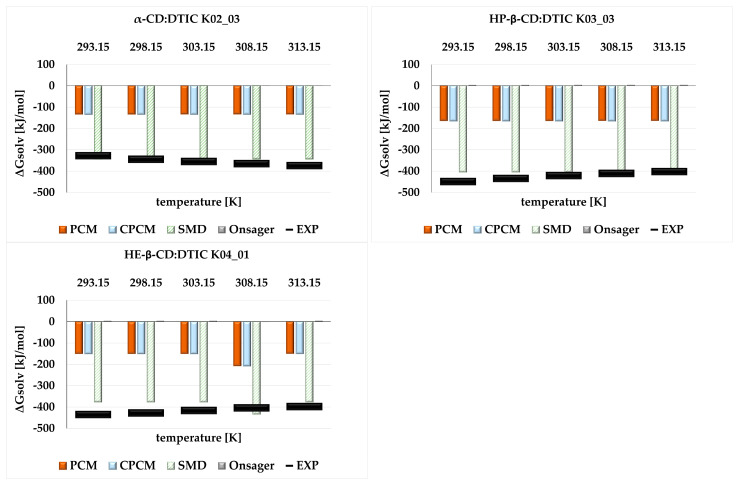
ΔG_solv_ [kJ/mol] obtained in the temperature range (293.15–313.15 K) from theoretical analysis for the most stable structures of each complex α-CD:DTIC K02_03, HP-β-CD:DTIC K03_03, and HE-β-CD:DTIC K04_01, and from conductometric measurements (EXP).

The advantage of the SMD model over the others is clearly evident in the analysis of the Gibbs free energy of solvation (ΔG_solv_), presented in Figure 7. This analysis again focused solely on the most stable structure from each complex. While all complexes demonstrated good solvation, the β-CD derivatives exhibited the highest solvation efficiency. Once again, the largest discrepancies were observed for the Onsager model, which surprisingly indicated positive values of ΔG_solv_.

A summary of the key physicochemical properties of the solvent used, such as density, viscosity, and dielectric constant, presented in Table S3, enabled the determination of the molar concentration of the analyzed solutions.

Data concerning dacarbazine molar conductivity (DTIC) in the presence of the analyzed cyclodextrins (α, HP-β, and HE-β) are presented in Tables S4–S6. The molar concentrations shown in the tables were calculated based on the relationships described in the publication [37]. The application of a new theoretical approach, presented in [37], allows for a more precise analysis of the process of formation of the inclusion complex, as well as the assessment of the theoretical conductivity. This enabled verification of the accuracy of these equations in the context of varying cyclodextrin molecular sizes and the properties of the complexed ions.

Complexation constants were determined based on electrolyte conductivity, using a formula developed and described in the author’s earlier publications [37,38,39]:

(2) $$K_{f}=\frac{\left(1-\alpha \right)}{\alpha [C_{CD}-\left(1-\alpha \right)C_{DTIC}]}$$

The molar conductivity of the tested solution is described by the following relationship:

(3) $$\Lambda_{obs}=\alpha \cdot \Lambda_{m}+(1-\alpha )\cdot \Lambda_{c}$$

The complex formation constant can be expressed using the following relationship:

(4) $$K_{f}=\frac{\left(\Lambda_{m}-\Lambda_{obs}\right)}{\left(\Lambda_{obs}-\Lambda_{c}\right)\cdot C_{CD}}$$

Using the above relationships, the concentration of cyclodextrin can be described by the equation:

(5) $$\left[C_{CD}\right]=C_{L}-\frac{C_{DTIC}(\Lambda_{m}-\Lambda_{obs})}{\left(\Lambda_{m}-\Lambda_{c}\right)}$$

Thus, the complexation constant can be represented by the following relationship:

(6) $$\Lambda =\left[K_{f}(c_{DTIC}-C_{CD}-1+\sqrt{K_{f}^{2}(C_{CD}-C_{DTIC}{)}^{2}+2K_{f}\left(C_{DTIC}+C_{CD}\right)+1}\right]\cdot \left[\frac{\Lambda_{DTIC}-\Lambda_{(DTIC)CD}}{2K_{f}C_{DTIC}}\right]+\Lambda_{(DTIC)CD}$$

where in Equations (5) and (6):

**C_L_**—total concentration of cyclodextrin in the solution, which includes both free cyclodextrin molecules and those bound to dacarbazine in the form of a complex;

Λ—molar conductivity of the solution prior to the addition of cyclodextrin;

Λ_(DTIC)CD_—molar conductivity of the solution (dacarbazine (DTIC) with added cyclodextrin);

Λ_c—_molar conductivity of the complexed ion;

C_Cd_—calculated current concentration of free cyclodextrin;

C_DTIC_—concentration of dacarbazine (DTIC) in solution.

The values of K_f_ and conductivity Λ_(DCTIC)CD_ are selected by minimizing the sum:

(7) $$\sum_{i=1}^{n}(\Lambda_{exp}-\Lambda_{calc}{)}^{2}$$

where

n—number of test solutions;

Λ_exp_—experimental molar conductivity calculated from equation;

Λ_calc—_molar conductivity calculated from equation;

Additionally, the values for Λ_DTIC_ and Λ_(DTIC)CD_ are described by the following dependencies:

(8) $$\Lambda_{DTIC}=\Lambda_{0DTIC}-{Sc_{DTIC}}^{\frac{1}{2}}+Ec_{DTIC}lnc_{DTIC}+J_{1}c_{S}+J_{2}c_{DTIC}.^{\frac{3}{2}}$$

(9) $$\Lambda_{(DTIC)CD}=\Lambda_{0(DTIC)CD}-{Sc_{DTIC}}^{\frac{1}{2}}+Ec_{DTIC}lnc_{DTIC}+J_{1}c_{S}+J_{2}c_{DTIC}.^{\frac{3}{2}}$$

In the course of this study, which aimed to evaluate theoretical and experimental models to describe the thermodynamic and structural properties of cyclodextrin complexes, special attention was paid to the investigation of interactions between dacarbazine and three types of cyclodextrin in water: α-cyclodextrin, hydroxypropyl-β-cyclodextrin, and hydroxyethyl-β-cyclodextrin. The experimental results on molar conductivity are presented in Tables S3–S5, where the dependence of molar conductivity values on concentration and temperature is analyzed.

The observations clearly indicate an increase in the molar conductivity as a function of both concentration and temperature. This behavior is fully consistent with the conductivity theory, which states that higher temperatures lead to increased ion mobility, resulting in higher conductivity. This phenomenon is a result of weak interactions between ions and the solvent at elevated temperatures, allowing the ions to move more freely.

In turn, when the concentration of the studied hosts, i.e., α-cyclodextrin and HP-β-CD, increased, a noticeable decrease in conductivity was observed. This phenomenon may result from the formation of inclusion complexes between the investigated dacarbazine and the cyclodextrins. The formation of such complexes leads to a reduction in the availability of free ions, which in turn contributes to the decrease in molar conductivity.

The results presented in Table 5 show the K_f_ values of the complex formation constants for dacarbazine, as well as the calculated limiting conductivities for the discussed with dacarbazine in water. For α-CD, the values of lnK_f_ were the following: 5.4657 (T = 288 K), 6.7995 (T = 293 K), 7.3238 (T = 298 K) 8.1127 (T = 303 K), and 9.074 (T = 308 K). A surprising observation was the nonlinear nature of the lnK_f_ change with temperature, with a distinct maximum observed at 303 K. This behavior differs from that in the other two cases for HP-β-CD, where lnK_f_ decreased linearly from 7.5536 (T = 288 K) to 2.27 (T = 308 K), and similarly for HE-β-CD, lnK_f_ decreased linearly from 6.3547 to 3.2456. The same phenomenon was also observed in the limiting conductivity values for the respective systems.

This unusual nonlinear lnK_f_ behavior in the case of α-CD suggests the presence of complex interaction mechanisms that may change with temperature. The appearance of a maximum around 303 K may indicate a shift in the system’s equilibrium possibly between two different complexation mechanisms. Increased flexibility of the α-CD molecule at this temperature may promote conformational reorganization, improving spatial fit to the dacarbazine molecule. This phenomenon was confirmed by computational analysis. The formation of the α-CD:DTIC complex was associated with a strong deformation, significantly greater than that observed for the HE-β-CD:DTIC complex. Additionally, a reduction in the hydration of both the host and the cyclodextrin may occur, facilitating inclusion. However, above 303 K, excessive increase in kinetic energy may disturb complex stability, for instance, by reducing the residence time of dacarbazine inside the cyclodextrin cavity or by increasing competition from water molecules.

In contrast, HP-β and HE-β, which are chemically modified cyclodextrins, exhibited a more linear decline in lnK_f_ with temperature. This suggests a more predictable, classical complexation mechanism, strongly dependent on temperature, and lacking additional conformational effects.

Dacarbazine, as a molecule with distinct lipophilic properties and a relatively rigid planar structure, shows a particular tendency to form complexes with cyclodextrins that possess a suitably matched internal geometry. Its hydrophobic nature favors less polar environments; thus, the inner, apolar cavity of a cyclodextrin provides a natural complexation site. In the case of α-CD, whose cavity is narrow and compact, the dacarbazine molecule may be in the size-fit limit, which explains the observed (lnK_f_) maximum at 303 K. This temperature may promote optimal structural fit and reduced hydration of both the guest and cyclodextrin, thereby facilitating inclusion. However, above this temperature, the excessive kinetic energy may destabilize the complex, either by reducing the residence time of dacarbazine within the cyclodextrin or increasing competition from water molecules.

For HP-β- and HE-β-cyclodextrins, the situation is more straightforward, as their modified cavities are larger and more polar, weakening the binding with dacarbazine due to reduced structural and energetic compatibility. As a result, a linear decline in lnK_f_ with increasing temperature was observed, typical of systems in which complexation weakens due to entropy-driven effects.

The temperature dependences of the complex formation constant were used to determine the free enthalpy of the complex formation.

(10) $$∆G\left(T\right)=-RTlnK_{f}(T)$$

The above relationship can be presented as follows.

(11) $$∆G\left(T\right)=A+BT+CT^{2}$$

The enthalpy values are presented as the first derivative of the free enthalpy after temperature at constant pressure.

(12) $$∆S^{o}=-(\frac{\partial ∆G^{o}}{\partial T}{)}_{p}=-B-2CT$$

The enthalpy is calculated from the following relationship:

(13) $$∆H^{0}=∆G^{o}+T∆S^{o}=A-CT^{2}\;\;\;\;\;\;\;$$

Based on experimental data and calculations derived from the constant of relationship between the complex formation K_f_ and temperature, the changes in enthalpy ΔH^0^, Gibbs free energy ΔG^0^, and entropy ΔS^0^ were determined for dacarbazine inclusion complexes with three cyclodextrins. Variations in these parameters, obtained from conductivity measurements, constitute a significant part of the thermodynamic analysis of the complex formation process.

The calculated ΔG^0^ values for each cyclodextrin indicate that the formation of inclusion complexes was most spontaneous for α-cyclodextrin, as evidenced by its strongly negative ΔG^0^ values. This suggests that, in this case, complex formation is the most energetically favourable. For HP-β-CD, the ΔG^0^ values were slightly less negative, which implies that the complexation process is less spontaneous but still thermodynamically feasible. In contrast, HE-β-CD exhibited the least negative ΔG^0^ values, suggesting that complex formation is the least spontaneous in this case. This may be due to the presence of substituents in the HE-β-CD structure, which weakens its ability to form complexes with dacarbazine anions.

All ΔH^0^ values were negative, indicating that complex formation processes were exothermic and associated with energy release. However, the enthalpy changes varied depending on the cyclodextrin. For α-cyclodextrin, the enthalpy values were the lowest, which means that the complexation process was the most energetically favourable. HP-β-CD exhibited higher, but still negative enthalpy values, confirming an exothermic process. The highest enthalpy changes were recorded for HE-β-CD, suggesting that the complex formation in this case involved greater energy release, possibly due to more intense dehydration during complex formation.

Entropy changes ΔS^0^ (Table S7) were also negative for all cyclodextrins, which is typical for inclusion complexation processes, where the molecular organization resulting from guest molecule in the cyclodextrin cavity leads to reduction of entropy. However, the change in entropy increases with rising temperature. For HE-β-CD, ΔS^0^ values were the highest, suggesting that complex formation involved greater molecular reorganization and more intense hydrophobic dehydration. The higher entropy in the case of HE-β-CD may also result from a better fit of the anion within the cyclodextrin cavity, allowing greater molecular distribution freedom. This phenomenon may be due to the more spacious structure of this cyclodextrin, which enables more flexible positioning of the phenolic anions inside the cavity.

The increase affected the complex formation process by increasing the kinetic energy of molecules, which facilitates their penetration into the cyclodextrin cavity and enhances the complexation process. The ΔG^0^ values became increasingly negative with increasing temperature, indicating that complex formation became more spontaneous. At higher temperatures, the anion molecules possess a greater kinetic energy, which enables better alignment within the cyclodextrin cavity. Therefore, higher temperatures favor more efficient molecular encapsulation and improve the effectiveness of complex formation.

From a thermodynamic theory perspective, the formation of an inclusion complex can be considered in terms of replacement of polar–nonpolar interactions (between water molecules and the drug or cyclodextrin) by nonpolar–nonpolar interactions between the cyclodextrin and its guest. This substitution leads to a reduction in system energy, promoting the formation of stable complexes. As a result, the complex formation process is exothermic and spontaneous, and changes in enthalpy and entropy are essential factors shaping this process.

In summary, thermodynamic calculations indicated that the inclusion complexation of dacarbazine in the presence of cyclodextrins was exothermic and its spontaneity increased with temperature. The values of enthalpy, free energy, and entropy suggest that the complex with HE-β-CD was the least spontaneous, probably due to the influence of substituents on its structure. These processes are thermodynamically favorable, especially for α-CD and HP-β-CD, and their behavior aligns with theoretical predictions regarding interactions between cyclodextrin molecules, their guests, and water.

It is important to note that the ionization behavior of dacarbazine (DTIC) may influence both theoretical modeling and interpretation of experimental data. Literature reports indicate that the hydrazine moiety of DTIC has a pKa value of approximately 3.5–4.5, suggesting possible partial ionization in aqueous solutions depending on pH. However, the exact pH of the solutions used in the conductometric measurements was not monitored during the experiments.

Due to the absence of pH data, the neutral (unionized) form of DTIC was selected for theoretical modeling as a practical approximation. This choice allowed for consistent comparison across all cyclodextrin complexes while keeping computational complexity manageable. Nevertheless, we acknowledge that a fraction of DTIC may exist in an ionized form under experimental conditions, which could contribute to the observed changes in molar conductivity.

While this introduces a degree of uncertainty in correlating theoretical and experimental results, the overall thermodynamic trends and comparative behavior between different cyclodextrin hosts remain valid. Future studies could benefit from pH-controlled conditions and modeling of alternative protonation states to refine the accuracy of the analysis.

## 3. Materials and Methods

### 3.1. Computational Analysis

The theoretical analysis was divided into two main stages. The first stage involved identifying the most energetically stable conformer of dacarbazine through a conformational search. The second stage focused on determining the most stable complex in each system—α-CD:DTIC, HP-β-CD:DTIC, and HE-β-CD:DTIC—via configurational search.

**Conformational Search of DTIC:** dacarbazine was modeled using the HyperChem program [40], based on the crystallographic structure retrieved from the Cambridge Structural Database (CSD; refcode: 1214885) [41]. Since the crystallographic structure lacked hydrogen atoms, they were added manually. Ten torsion angles were defined in the molecule, as illustrated in Figure S6. With this setup, the conformational search was initiated using the HyperChem tool -conformational search.

During the conformational search, a random number (from 1 to 10) of torsion angles were modified within a defined range—0–180° for acyclic and 0–120° for cyclic torsions. Each resulting conformer was optimized using the AMBER99 force field [42,43], chosen based on its successful application in our previous studies on compounds structurally similar to DTIC [32,44,45,46,47]. Furthermore, AMBER99 is suitable for systems containing sugar units, which is relevant to our complexation studies with cyclodextrins (CDs).

A total of 100 distinct conformers were generated and subsequently refined using semiempirical methods—PM6-D3-H4 [48] and PM7 [49]—in MOPAC2016 [50]. These optimizations were performed in water using the conductor-like screening model (COSMO) [51]. The optimized structures were then subjected to further geometry optimization at the density functional theory (DFT) level using Gaussian16 (Rev. C.02) [52], with water described via the polarizable continuum model (PCM) [7]. Several DFT methods were tested, including M08-HX-D3/aug-cc-pVTZ [53,54,55], M08-HX-D3/6-31G(d,p) [53,56], DSD-PBEP86-D3BJ/aug-cc-pVTZ [57,58,59], and B97-D3/aug-cc-pVTZ [59]. These methods are widely recommended for studying organic compounds, non-covalent interactions, and—especially in the case of M08-HX-D3—thermodynamic properties [60,61]. Since D3 dispersion corrections are not natively implemented for M08-HX in Gaussian16, the IOp keywords (3/174–176) were used following the guidance in ref. [60], with S6 and SR6 set to 1.0000 and 1.6247, respectively.

For all DFT-optimized structures, harmonic vibrational frequency analyses were performed to confirm that the structures represent local minima on the potential energy surface. These calculations also provided values for enthalpy (H) and Gibbs free energy (G). Entropy corrections for low-frequency vibrational modes (<100 cm^−1^) were applied using the Grimme approach, as implemented in the GoodVibes program [62].

**Configurational Search of CDs:DTIC Complexes:** the procedure used to identify the most stable CD:DTIC complexes closely mirrored that applied to isolated DTIC. For cyclodextrins, we did not perform the conformational search, but we used the coordinates of the experimental structures. Coordinates of α-CD and β-CD were taken from the CSD (α-CD: CHXAMH02 [63]; β-CD: BCDEXD05 [64]). Crystallographic water molecules were removed prior to modeling. Since HP-β-CD and HE-β-CD had not been crystallized, their structures were built by modifying β-CD. For HP-β-CD, we followed recommendations from literature [65], which indicated that the most preferred form includes four hydroxypropyl groups attached to the primary rim. For HE-β-CD, no specific conformational guidance was found, so hydroxyethyl groups were added in the same positions as in HP-β-CD. A schematic representation of both modified CDs is provided in Figure 1. All cyclodextrins were optimized at the M08-HX-D3/6-31G(d,p) theory level in water described by four theoretical models (PCM, CPCM, SMD and Onsager). The use of experimental structures of CDs in our analysis stems from the results obtained in our previous study, in which β-cyclodextrin complexes with loratadine were analyzed [46]. The experimental β-cyclodextrin molecule formed significantly more stable complexes than the theoretical model.

Initial models of each complex were built by placing DTIC in various orientations relative to the CD cavities, as shown in Figure S4. A total of four distinct starting configurations were generated for each complex. For each configuration, a systematic rotation of DTIC around the X, Y, and Z axes was performed in 20° increments, resulting in 1457 structures per system. These were initially optimized in HyperChem using the AMBER99 force field.

Subsequent optimizations were performed using the PM7 method in MOPAC2016. The resulting complexes were ranked by their heats of formation (H_f_), and the three lowest-energy structures (01-03) from each orientation set (K01–K04) for every CD:DTIC system were selected for further analysis.

Final optimizations were carried out at the DFT level using M08-HX-D3/6-31G(d,p), a method recommended for thermochemical studies.

Each complex was optimized in water using four different implicit solvent models: PCM [7], CPCM [10,66], SMD [13], and Onsager [4,6]. In this case, harmonic vibrational frequency calculations were also performed, and the absence of imaginary frequencies confirms that the obtained molecules correspond to true energy minima on the potential energy surface. Additionally, G values were corrected using the GoodVibes program.

The stability of the complexes was estimated by calculating the complexation (E_compl_) energy using the supramolecular approach as follows:

(14) $$E_{compl}=E_{complex}^{OPT}-\left(E_{CD}^{OPT}+E_{DTIC}^{OPT}\right)$$

where

${\;E}_{complex}^{OPT}$ —energy of the optimized complex;

$E_{CD}^{OPT}$ and ${\;E}_{DTIC}^{OPT}—$energies of CDs and DTIC, respectively, in their most stable geometries.

**Preparation of Explicit Solvent Model:** in HyperChem, a periodic box (20 × 20 × 20 Å) containing 265 water molecules was built around the system. The entire setup was first optimized at the molecular mechanics level using the AMBER99 force field. Following optimization, only the 20 water molecules closest to the solute were retained; the remaining, more distant water molecules were removed.

The resulting 20-water cluster system was then reoptimized using the PM7 method with the COSMO solvent model. Finally, the system was further refined at the DFT level using the M08-HX-D3/6-31G(d,p) method with the PCM implicit solvent model.

### 3.2. Materials

Three cyclodextrins (α-CD, HP-β-CD, and HE-β-CD) and dacarbazine were used for the conductometric measurements. As reported by Merck, the average degree of substitution for HP-β-CD was 0.5–1.3 hydroxypropyl (–C_3_H_7_O) groups per glucose unit. In the case of HE-β-CD, Merck does not provide information regarding the degree of substitution or the average molecular mass. Therefore, the information for HE-β-CD shown in Table 6, which details the chemical specifications, was based on the theoretical model presented in Figure 1. Water was used as the solvent. All reagents used were of high purity.

### 3.3. Electrical Conductivity Estimation—Exploratory Method

Electrical conductivity estimates were performed agreeing to the strategies (see [67,68,69]). The Wayne-Kerr 6430B RLC bridge was utilized, with an estimation instability of 0.02%. The estimation setup included a three-electrode conductivity cell made of sodium-Is free glass, as depicted in [70]. The gear was carried out employing a reference arrangement of KCl with exceptionally high virtue (Merck, 99.999%) [71]. Temperature control during the tests was guaranteed by the BU 20F indoor regulator (Lauda, Germany), in conjunction with the DLK 25 stream cooling framework from the same company. The temperature stability of the framework did not exceed 0.005 K. Temperature estimations were performed using the Amarell 3000TH Advertisement thermometer (Amarell, Germany). The tests were carried out over a recurrence run from 0.2 to 20 kHz, at the following frequencies: 0.2, 0.5, 1.0, 1.5, 2.0, 3.0, 5.0, 10.0, and 20.0 kHz. Tables S4 and S5 give estimates of standard vulnerabilities. The total instability, counting components such as the calibration of the estimation cell and the virtue of the reagents, was roughly ±0.05%. A nitty-gritty depiction of the strategy can be found in reference [72]. All arrangements were gravimetrically arranged using an explanatory adjustment (Sartorius RC 210D) with a precision of ±1∙10^−5^ g. The explanatory method was based on the strategy proposed by Bešter-Rogačeta [73,74]. The temperature run explored was from 293.15 to 313.15 K, with 5 K interims. On the basis of the collected information, restricting molar conductivities, affiliation constants, and thermodynamic functions for the examined cyclodextrins with dacarbazine in water were calculated.

## 4. Conclusions

In this work, we investigated the impact of different implicit solvent models (PCM, CPCM, SMD, and Onsager) on the geometrical and thermodynamic properties of inclusion complexes. The study focused on dacarbazine, an anticancer drug, in combination with three types of cyclodextrins: α-cyclodextrin (α-CD), hydroxypropyl-β-cyclodextrin (HP-β-CD), and hydroxyethyl-β-cyclodextrin (HE-β-CD). Additionally, an explicit solvent model, in which water molecules were directly added to the system, was also explored.

Our results confirmed that cyclodextrins form stable inclusion complexes with dacarbazine, and the stability of these complexes depends on both the type of cyclodextrin and temperature. Among the studied hosts, HE-β-CD formed the most stable complexes, while the smallest cyclodextrin, α-CD, demonstrated a stability comparable to HP-β-CD. Implicit solvent models showed minimal impact on the geometry of the complexes. In contrast, the explicit solvent model, which involved the addition of 20 water molecules, had a significant effect on complex geometry. This influence is attributed to the formation of hydrogen bonds between the solute and solvent molecules, which distort the solvated structure.

From a thermodynamic perspective, the analysis of parameters such as enthalpy (ΔH), Gibbs free energy (ΔG), entropy (ΔS), and Gibbs free energy of solvation ΔG_solv_ indicated that the SMD model best reproduced experimental values. Therefore, the SMD model is recommended for studying systems similar to those analyzed in this work.

Notably, for the α-CD complex, a nonlinear relationship between ln(K_f_) and temperature was observed, with a maximum at 303 K. This suggests a complexation mechanism likely involving conformational reorganization of the cyclodextrin and optimal geometric matching at this temperature. In contrast, HP-β-CD and HE-β-CD, both chemically modified, exhibited a linear decrease in ln(K_f_) with increasing temperature, indicative of a more classical complexation mechanism primarily governed by entropic and energetic factors.

Further thermodynamic analysis confirmed that the complexation process was both spontaneous and exothermic. The greatest energy gain and most negative ΔG and ΔH values were recorded for complexes with unmodified α-CD. Moreover, the observed decrease in entropy with increasing molar mass of the cyclodextrins and their anions suggest that dehydration of hydrophobic fragments plays a key role in the inclusion process. This supports the conclusion that complex formation is mainly driven by enthalpic contributions arising from the replacement of solute–solvent interactions with more energetically favorable interactions within the cyclodextrin cavity.

## Figures and Tables

**Table 1:** RMSD values calculated with respect to the structures obtained from PCM calculations and presented in Figure 4.

Molecule	CPCM	SMD	Onsager
α-CD:DTIC			
K02_03	0.00236	0.04637	0.05331
K01_01	0.00417	0.04407	0.03490
K03_03	0.00302	0.01402	0.01769
HP-β-CD:DTIC			
K03_03	0.00433	0.46008	0.08733
K04_03	0.00899	0.64110	0.15543
K04_01	0.00670	0.33926	0.27704
HE-β-CD:DTIC			
K04_01	0.00674	0.93252	0.10769
K03_02	0.00422	0.44491	0.31047
K03_03	0.02785	0.60237	0.78544

**Table 2:** ΔH and ΔG [kJ/mol] in the temperature range 293.15–313.15 K calculated for the α-CD:DTIC complexes presented in Figure 4. Experimental values (EXP) are written in blue.

293.15										
α-CD:DTIC	ΔH PCM	ΔH CPCM	ΔH SMD	ΔH Onsager	ΔH EXP	ΔG PCM	ΔG CPCM	ΔG SMD	ΔG Onsager	ΔG EXP
K01_01	−94.38	−93.65	−92.68	−127.44	−91.32	−18.27	−17.98	−15.01	−50.49	-17.85
K03_03	−92.22	−91.75	−92.41	−96.74		−17.17	−16.95	−18.46	−22.39	
298.15										
K01_01	−94.41	−93.68	−92.68	−127.45	−94.72	−17.02	−16.74	−13.77	−49.23	−20.11
K03_03	−92.21	−91.75	−92.38	−96.72		−15.95	−15.73	−17.26	−21.18	
303.15										
K01_01	−94.44	−83.79	−92.68	−127.45	−101.52	−15.77	−16.57	−12.53	−47.99	−22.87
K03_03	−92.20	−91.74	−92.34	−96.70		−14.72	−14.51	−16.07	−19.97	
308.15										
K01_01	−94.42	−93.74	−92.67	−127.39	−113.65	−14.54	−14.26	−11.30	−45.66	−17.42
K03_03	−92.16	−91.73	−92.30	−96.68		−13.51	−13.29	−14.88	−18.76	
313.15										
K01_01	−94.49	−95.51	−92.67	−127.46	−121.23	−13.28	−12.28	−10.07	−45.49	−14.23
K03_03	−92.13	−91.72	−92.27	−96.66		−12.88	−12.06	−13.69	−17.55	

**Table 3:** ΔH and ΔG [kJ/mol] in the temperature range 293.15–313.15 K calculated for the HP-β-CD:DTIC complexes presented in Figure 4. Experimental values (EXP) are written in blue.

293.15										
HP-β-CD:DTIC	ΔH PCM	ΔH CPCM	ΔH SMD	ΔH Onsager	ΔH EXP	ΔG PCM	ΔG CPCM	ΔG SMD	ΔG Onsager	ΔG EXP
K04_01	−82.89	−82.75	−82.53	−118.53	−104.62	−3.86	−4.72	6.16	−34.05	−18.51
K04_03	−92.88	−92.72	−109.50	−119.03		−16.49	−16.05	−27.31	−41.87	
298.15										
K04_01	−82.93	−82.82	−82.57	−118.57	−109.12	−2.59	−3.01	7.54	−32.71	−15.27
K04_03	−92.91	−92.76	−109.54	−119.06		−15.22	−15.75	−25.97	−40.61	
303.15										
K04_01	−82.97	−82.86	−82.61	−112.98	−113.67	−1.31	−1.74	8.92	−29.73	−12.36
K04_03	−92.94	−92.80	−109.58	−113.45		−13.95	−14.49	−24.63	−37.70	
308.15										
K04_01	−83.01	−82.90	−82.64	−118.63	−118.02	−0.04	−0.80	10.31	−30.03	−9.06
K04_03	−92.97	−92.83	−109.61	−119.09		−12.69	−13.25	−23.28	−38.07	
313.15										
K04_01	−83.04	−82.93	−82.68	−118.66	−121.51	1.23	0.79	11.69	−28.68	−5.91
K04_03	−93.00	−92.85	−109.65	−119.10		−11.42	−11.98	−21.94	−36.80	

**Table 4:** ΔH and ΔG [kJ/mol] in the temperature range 293.15–313.15 K calculated for the HE-β-CD:DTIC complexes presented in Figure 4. Experimental values (EXP) are written in blue.

293.15										
HE-β-CD:DTIC	ΔH PCM	ΔH CPCM	ΔH SMD	ΔH Onsager	ΔH EXP	ΔG PCM	ΔG CPCM	ΔG SMD	ΔG Onsager	ΔG EXP
K03_02	−149.55	−149.66	−169.32	−196.69	−156.20	−60.99	−60.92	−75.34	−105.44	−15.49
K03_03	−149.19	−148.66	−156.29	−155.99		−61.27	−59.47	−62.91	−68.11	
298.15										
K03_02	−149.64	−149.75	−169.43	−196.78	−149.50	−59.56	−59.50	−73.84	−103.98	−13.25
K03_03	−149.28	−148.76	−156.37	−156.06		−59.86	−58.05	−61.42	−66.70	
303.15										
K03_02	−149.72	−149.83	−169.54	−196.86	−133.60	−58.13	−58.07	−72.35	−102.53	−11.43
K03_03	−149.37	−148.86	−156.46	−156.13		−58.45	−56.62	−59.94	−65.30	
308.15										
K03_02	−148.16	−151.56	−169.64	−196.94	−127.50	−54.43	−58.92	−70.85	−101.07	−9.79
K03_03	−147.82	−150.59	−156.54	−156.19		−54.76	−57.47	−58.46	−63.89	
313.15										
K03_02	−149.88	−149.99	−169.74	−196.20	−116.80	−55.28	−55.21	−69.36	−100.58	−8.45
K03_03	−149.55	−149.03	−156.62	−156.25		−55.63	−53.76	−56.98	−62.27	

**Table 5:** The value of constant formation *K_f_* [dm^3^/mol], theoretical conductivity Λ_CD(DTCI)_ [S∙cm^2^/mol^−1^] for α-CD, HE-β-CD, and HP-β-CD with the dacarbazine (DTIC).

α-CD					HE-β-CD			
T [K]	K_f_ [dm^3^/mol]	lnK_f_ [dm^3^/mol]	Λ_CD(DTIC)_ [S∙cm^2^/mol^−1^]	σ(Λ)	K_f_ [dm^3^/mol]	lnK_f_ [dm^3^/mol]	Λ_CD(DTIC)_ [S∙cm^2^/mol^−1^]	σ(Λ)
293.15	1516 ± 3	7.3238	58,76 ± 0.01	0.01	575 ± 6	6.3547	61.04 ± 0.02	0.01
298.15	3337 ± 2	8.1127	65.55± 0.01	0.02	209 ± 4	5.3433	67.83 ± 0.01	0.02
303.15	8726 ± 4	9.0740	70.65 ± 0.01	0.01	93 ± 2	4.5345	72.93 ± 0.01	0.01
308.15	897 ± 5	6.7995	79.88 ± 0.02	0.02	46 ± 1	3.8200	82.16 ± 0.02	0.01
313.15	236 ± 2	5.4657	85.87 ± 0.02	0.02	26 ± 1	3.2456	88.15 ± 0.01	0.02
HP-β-CD								
T [K]	K_f_ [dm^3^/mol]	lnK_f_ [dm^3^/mol]		Λ_CD(DTIC)_ [S∙cm^2^/mol^−1^]		σ(Λ)		
293.15	1907 ± 7	7.5536		64.86 ± 0.02		0.01		
298.15	473 ± 5	6.1602		67.83 ± 0.02		0.01		
303.15	135 ± 4	4.9040		72.93 ± 0.01		0.01		
308.15	34 ± 2	3.5442		82.16 ± 0.02		0.02		
313.15	9.7 ± 2	2.2700		88.15 ± 0.01		0.01		

**Table 6:** Specification of chemical samples. A schematic representation of the molecules is shown in Figure 1.

Chemical Name	Chemical Formula	Source	CAS Number	Purity	Molecular Mass [g/mol]
DTIC	C_6_H_10_N_6_O	Merck	4342-03-4	≥0.999	182.18
α-CD	C_36_H_60_O_30_	TCL	10016-20-3	≥0.998	972.84
HP-β-CD	C_42_H_70_O_35_·(C_3_H_7_O)_n_	Merck	128446-35-5	≥0.998	1396.00 *
HE-β-CD	C_42_H_70_O_35_·(C_3_H_7_O)_4_	Merck	128446-32-2	≥0.998	1315.23

* Average value presented by Merck.

## Data Availability

The original contributions presented in this study are included in the article. Further inquiries can be directed to the corresponding author(s).

## Supplementary Materials

The following supporting information can be downloaded at https://www.mdpi.com/article/10.3390/molecules30112309/s1. The following supported information are available online: Figures S1–S6; Schematic representation of torsion angles changed to compute the rotational energy profile; Relative energy differences for DTIC; DTIC-1 and α-CD:DTIC in the presence of 20 water molecules; Initial model of complexes; Torsion angles marked in DTIC. Tables S1–S7.; Total energy values of complexes; Coordinates of complexes; Densities of complexes.; Molar concentrations of complexes; Entropy values of complexes.
