# Supplementary material for: Evaluating Theoretical Solvent Models for Thermodynamic and Structural Descriptions of Dacarbazine–Cyclodextrin Complexes. The Theoretical and Conductometric Study
Source: Molecules. 2025 May 24;30(11):2309. doi: 10.3390/molecules30112309 (PMC12156229; doi:10.3390/molecules30112309)
Supplement: Supplementary file 1 [file molecules-30-02309-s001.zip › molecules-3617222-supplementary.pdf]

# Evaluating Theoretical Solvent Models for Thermodynamic and Structural Descriptions of Dacarbazine–Cyclodextrin Complexes. The Theoretical and Conductometric Study

**Zdzisław Kinart *, Marta Hoelm * and Martyna Imińska**

Department of Physical Chemistry, Faculty of Chemistry, University of Lodz, Pomorska 163/165, 90-236 Lodz, Poland; martyna.iminska@edu.uni.lodz.pl

* Correspondence: zdzislaw.kinart@chemia.uni.lodz.pl (Z.K.); marta.hoelm@chemia.uni.lodz.pl (M.H.)

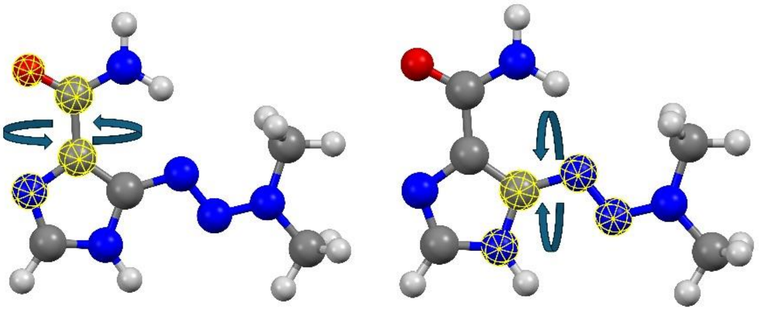


**Figure S1.** Schematic representation of torsion angles (highlighted in yellow) scanned from 0° to 360° in 10° increments to compute the rotational energy profile and determine the barrier ($\Delta E_{rot}$) for the carboxamide and 3,3-dimethyltriaz-1-en-1-yl groups.

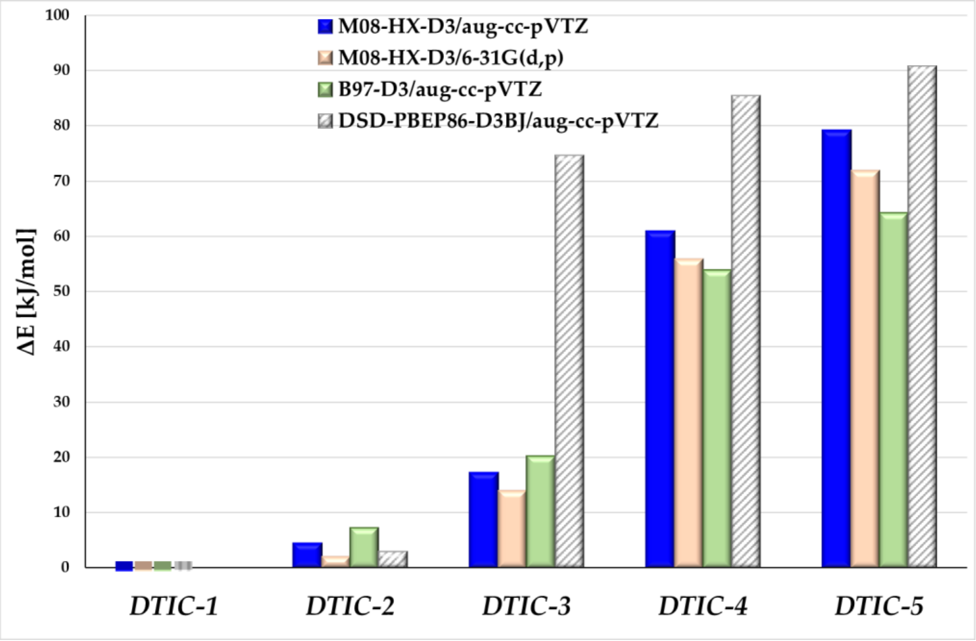


**Figure S2.** The relative energy differences (ΔE, in kJ/mol) between DTIC-1 and four other, less stable conformers obtained at the M08-HX-D3/aug-cc-pVTZ, M08-HX-D3/6-31G(d,p), B97-D3/aug-cc-pVTZ, and DSD-PBEP86-D3BJ/aug-cc-pVTZ theory levels.

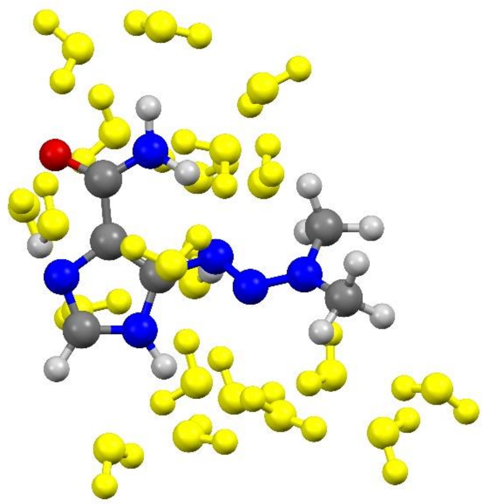

**Figure S3.** DTIC-1 in the presence of twenty water molecules (highlighted in yellow; DTIC-1 (20 $H_2O$)). The structure was optimized at the M08-HX-D3/aug-cc-pVTZ theory level.

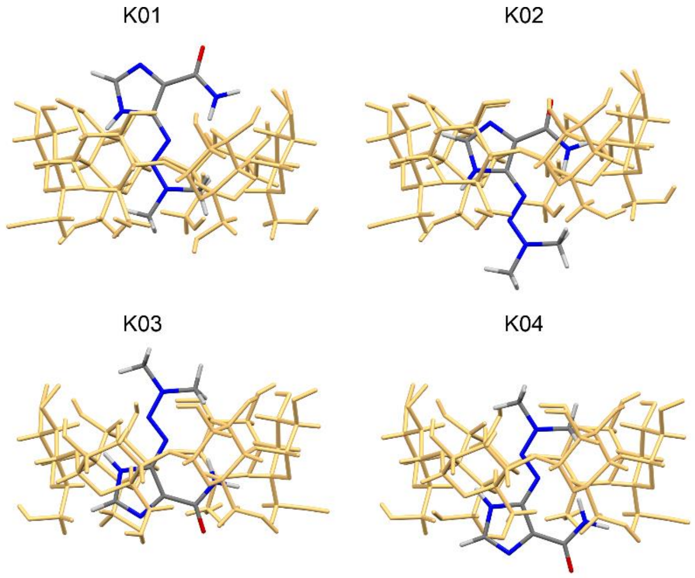


**Figure S4.** Initial models of complex CDs:DTIC. Cyclodextrin is highlighted in dark yellow.

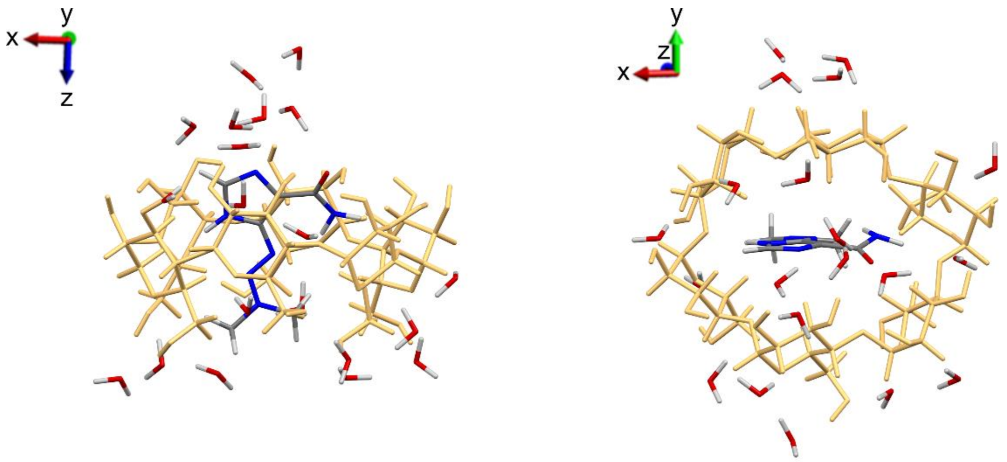


**Figure S5.** The most stable $\alpha$-CD:DTIC complex, K02_03, obtained from calculations performed at the M08-HX-D3/6-31G(d,p) theory level in the presence of twenty explicitly added water molecules. Cyclodextrin is highlighted in dark yellow.

**Table S1.** Total energy values [Hartree] obtained at the M08-HX-D3/6-31G(d,p) theory level in water (PCM) for the most stable complexes presented in Figure 4 of the main article.

| **Molecule** | **Energy** |
|---|---|
| α-CD:DTIC K02_03 | -4301.6460 |
| α-CD:DTIC K01_01 | -4301.6394 |
| α-CD:DTIC K03_03 | -4301.6382 |
| HP-β-CD:DTIC K03_03 | -5684.6240 |
| HP-β-CD:DTIC K04_03 | -5684.6202 |
| HP-β-CD:DTIC K04_01 | -5684.6175 |
| HE-β-CD:DTIC K04_01 | -5527.3902 |
| HE-β-CD:DTIC K03_02 | -5527.3794 |
| HE-β-CD:DTIC K03_03 | -5527.3792 |

**Table S2.** Coordinates [Å] of the most stable complexes, presented in Figure 4 of the main article, obtained at the M08-HX-D3/6-31G(d,p) theory level in water (PCM).

| **$\alpha$-CD:DTIC** | | | | | | | | | |
|---|---|---|---|---|---|---|---|---|---|
| **Atomic Number** | **K02_03** | | | **K01_01** | | | **K03_03** | | |
| | **x** | **y** | **z** | **x** | **y** | **z** | **x** | **y** | **z** |
| 6 | -5.6569 | 0.1832 | 0.2741 | -5.0105 | -2.3067 | 0.7305 | 0.3650 | -4.5145 | -1.1463 |
| 6 | -5.9616 | 1.1842 | -0.8356 | -5.8512 | -1.7150 | -0.3973 | -0.5302 | -4.0515 | -2.2901 |
| 6 | -4.7543 | 2.0889 | -1.0402 | -5.3208 | -0.3262 | -0.7216 | -1.4493 | -2.9600 | -1.7562 |
| 6 | -4.1849 | 2.6678 | 0.2579 | -5.1809 | 0.5587 | 0.5195 | -2.2629 | -3.5105 | -0.5901 |
| 6 | -4.2098 | 1.6682 | 1.4237 | -4.6225 | -0.2117 | 1.7217 | -1.3255 | -4.0932 | 0.4811 |
| 6 | -4.1424 | 2.3622 | 2.7752 | -4.8081 | 0.4997 | 3.0465 | -2.0434 | -4.8864 | 1.5766 |
| 6 | -2.6350 | -4.1621 | -0.1856 | -0.3021 | -4.6348 | 0.1234 | 5.0499 | -2.2920 | -0.4357 |
| 6 | -3.6397 | -3.9468 | -1.3213 | -1.3079 | -4.7782 | -1.0211 | 4.7483 | -3.3147 | -1.5323 |
| 6 | -4.0356 | -2.4808 | -1.3650 | -2.2007 | -3.5463 | -1.0234 | 3.2459 | -3.3383 | -1.7859 |
| 6 | -4.4844 | -1.9752 | -0.0003 | -2.8933 | -3.4567 | 0.3263 | 2.4833 | -3.6055 | -0.4913 |
| 6 | -3.4873 | -2.3528 | 1.0978 | -1.8280 | -3.3725 | 1.4251 | 2.9050 | -2.5989 | 0.5736 |
| 6 | -4.0490 | -2.1074 | 2.4887 | -2.4429 | -3.3505 | 2.8118 | 2.3514 | -2.8968 | 1.9557 |

| | | | | | | | | | |
|---|---|---|---|---|---|---|---|---|---|
| 6 | 2.6106 | -4.1184 | -0.5312 | 4.4366 | -2.5505 | -0.7120 | 4.2818 | 2.7446 | 0.4816 |
| 6 | 1.9073 | -3.9729 | -1.8904 | 3.5859 | -2.7461 | -1.9736 | 5.1718 | 2.5905 | -0.7523 |
| 6 | 0.4985 | -3.4010 | -1.7384 | 2.1024 | -2.6458 | -1.6068 | 4.9440 | 1.2083 | -1.3226 |
| 6 | -0.2531 | -4.1130 | -0.6223 | 1.8079 | -3.6286 | -0.4851 | 5.2270 | 0.1245 | -0.2907 |
| 6 | 0.5583 | -4.0882 | 0.6697 | 2.6938 | -3.3303 | 0.7229 | 4.5954 | 0.4154 | 1.0863 |
| 6 | -0.1129 | -4.8423 | 1.8036 | 2.4888 | -4.3600 | 1.8235 | 5.3598 | -0.2336 | 2.2417 |
| 6 | 5.8417 | -0.2310 | 0.6619 | 4.8879 | 2.1482 | 0.6653 | -0.4266 | 4.7313 | -0.6716 |
| 6 | 6.2839 | -1.1907 | -0.4490 | 5.6884 | 1.5805 | -0.5525 | 0.4085 | 4.3424 | -1.8981 |
| 6 | 5.0815 | -2.0439 | -0.8622 | 6.2601 | 0.1880 | -0.2472 | 1.3784 | 3.2277 | -1.5049 |
| 6 | 4.2801 | -2.5805 | 0.3359 | 5.2790 | -0.6997 | 0.5219 | 2.1725 | 3.6350 | -0.2707 |
| 6 | 4.1618 | -1.6393 | 1.5400 | 4.5900 | 0.0602 | 1.6743 | 1.2357 | 4.0630 | 0.8591 |
| 6 | 3.8743 | -2.4083 | 2.8342 | 4.7795 | -0.6392 | 3.0126 | 1.9884 | 4.6036 | 2.0642 |
| 6 | 2.7567 | 4.1134 | 0.2539 | 0.2366 | 4.6728 | 0.5010 | -5.1285 | 2.4448 | -0.0796 |
| 6 | 3.8355 | 4.0297 | -0.8309 | 1.1972 | 4.8934 | -0.6773 | -4.8585 | 3.5721 | -1.0798 |
| 6 | 4.3203 | 2.5881 | -0.9577 | 2.0794 | 3.6585 | -0.8087 | -3.3563 | 3.6471 | -1.3575 |
| 6 | 4.6905 | 1.9493 | 0.3726 | 2.7926 | 3.3743 | 0.4981 | -2.5597 | 3.7822 | -0.0658 |
| 6 | 3.5582 | 2.1700 | 1.3816 | 1.7579 | 3.2205 | 1.6113 | -2.9631 | 2.6524 | 0.8785 |
| 6 | 3.9171 | 1.7869 | 2.8094 | 2.3796 | 3.0595 | 2.9857 | -2.3075 | 2.7462 | 2.2374 |
| 6 | -2.4630 | 4.2823 | -0.3349 | -4.5467 | 2.7672 | -0.3227 | -4.3805 | -2.6931 | 0.2015 |
| 6 | -1.6982 | 4.2415 | -1.6641 | -3.7553 | 2.9286 | -1.6272 | -5.2428 | -2.3916 | -1.0228 |
| 6 | -0.3689 | 3.5186 | -1.4873 | -2.2585 | 2.8624 | -1.3180 | -5.0197 | -0.9458 | -1.4318 |
| 6 | 0.4249 | 4.1663 | -0.3661 | -1.9211 | 3.8518 | -0.2078 | -5.3283 | 0.0185 | -0.2906 |
| 6 | -0.4339 | 4.1569 | 0.9057 | -2.8665 | 3.7496 | 0.9948 | -4.6835 | -0.4403 | 1.0354 |
| 6 | 0.2231 | 4.8700 | 2.0744 | -2.6706 | 4.8914 | 1.9784 | -5.3766 | 0.0976 | 2.2880 |
| 1 | -6.5063 | -0.4876 | 0.4702 | -5.3170 | -3.3302 | 0.9956 | 1.0358 | -5.3275 | -1.4627 |
| 1 | -6.8360 | 1.7837 | -0.5288 | -6.9013 | -1.6560 | -0.0629 | -1.1297 | -4.9111 | -2.6402 |
| 1 | -3.9598 | 1.4615 | -1.4655 | -4.3011 | -0.4549 | -1.1270 | -0.8085 | -2.1491 | -1.3689 |
| 1 | -4.3103 | 3.3587 | -2.4258 | -5.6317 | 0.8717 | -2.1956 | -3.1234 | -2.8199 | -2.7447 |
| 1 | -4.7559 | 3.5764 | 0.5245 | -6.1553 | 1.0253 | 0.7544 | -2.9346 | -4.3093 | -0.9577 |
| 1 | -3.3364 | 0.9948 | 1.3263 | -3.5441 | -0.3242 | 1.5503 | -0.7643 | -3.2540 | 0.9366 |
| 1 | -3.1879 | 2.9008 | 2.8569 | -4.3921 | 1.5143 | 2.9685 | -2.7651 | -5.5760 | 1.1096 |
| 1 | -5.0490 | 0.9548 | 3.7230 | -4.4979 | -1.0592 | 4.1326 | -2.0745 | -3.7207 | 3.1134 |
| 1 | -4.9616 | 3.0995 | 2.8470 | -5.8840 | 0.5846 | 3.2806 | -1.2823 | -5.5047 | 2.0808 |
| 1 | -2.3794 | -5.2266 | -0.0652 | 0.3578 | -5.5136 | 0.2056 | 6.1146 | -2.3016 | -0.1474 |
| 1 | -4.5290 | -4.5719 | -1.1281 | -1.9218 | -5.6794 | -0.8491 | 5.0623 | -4.3053 | -1.1509 |
| 1 | -3.5874 | -4.0424 | -3.2511 | -1.2376 | -4.8827 | -2.9522 | 5.0292 | -3.4804 | -3.4127 |
| 1 | -3.1199 | -1.9271 | -1.6446 | -1.5560 | -2.6496 | -1.1168 | 2.9504 | -2.3393 | -2.1493 |
| 1 | -5.2983 | -1.3714 | -2.3384 | -3.9069 | -3.1410 | -1.8954 | 2.1594 | -4.0726 | -3.2173 |
| 1 | -5.4828 | -2.3725 | 0.2482 | -3.5308 | -4.3413 | 0.4986 | 2.6762 | -4.6310 | -0.1270 |
| 1 | -2.5549 | -1.7766 | 0.9612 | -1.2139 | -2.4672 | 1.2752 | 2.5669 | -1.5973 | 0.2522 |
| 1 | -3.2767 | -2.3630 | 3.2352 | -1.6355 | -3.3467 | 3.5636 | 2.6227 | -2.0594 | 2.6303 |
| 1 | -5.0132 | -3.7726 | 2.5302 | -2.8244 | -5.2415 | 2.8677 | 3.7935 | -4.1009 | 2.3588 |
| 1 | -4.2972 | -1.0403 | 2.6015 | -3.0230 | -2.4219 | 2.9277 | 1.2544 | -2.9518 | 1.9222 |
| 1 | 3.5180 | -4.7325 | -0.6384 | 5.4964 | -2.7671 | -0.9241 | 4.3968 | 3.7458 | 0.9311 |
| 1 | 1.8312 | -4.9636 | -2.3612 | 3.7876 | -3.7462 | -2.3880 | 6.2207 | 2.7058 | -0.4266 |
| 1 | 2.6183 | -2.2536 | -2.4207 | 4.0482 | -0.9488 | -2.4875 | 5.3807 | 3.4092 | -2.4875 |

| | | | | | | | | | |
|---|---|---|---|---|---|---|---|---|---|
| 1 | 0.5873 | -2.3361 | -1.4287 | 1.9184 | -1.6364 | -1.2125 | 3.8765 | 1.1466 | -1.6059 |
| 1 | -1.1334 | -3.5332 | -2.7742 | 0.7659 | -3.6729 | -2.6025 | 5.5950 | 0.1790 | -2.8168 |
| 1 | -0.4462 | -5.1597 | -0.9165 | 1.9913 | -4.6631 | -0.8312 | 6.3206 | 0.0120 | -0.1719 |
| 1 | 0.7397 | -3.0383 | 0.9626 | 2.4540 | -2.3155 | 1.0890 | 3.5410 | 0.0868 | 1.0573 |
| 1 | 0.5375 | -4.7937 | 2.6942 | 3.1309 | -4.1157 | 2.6856 | 4.7979 | -0.0466 | 3.1747 |
| 1 | 0.4385 | -6.5778 | 1.1603 | 3.6255 | -5.6739 | 0.9820 | 6.5761 | 1.2468 | 2.3660 |
| 1 | -1.0692 | -4.3595 | 2.0474 | 1.4430 | -4.3206 | 2.1599 | 5.4324 | -1.3165 | 2.1039 |
| 1 | 6.6773 | 0.3968 | 1.0063 | 5.2298 | 3.1623 | 0.9178 | -1.0804 | 5.5906 | -0.8879 |
| 1 | 7.0863 | -1.8366 | -0.0623 | 6.5446 | 2.2550 | -0.7316 | 0.9853 | 5.2309 | -2.2196 |
| 1 | 6.2147 | 0.3052 | -1.6797 | 4.3987 | 2.3144 | -1.8221 | -1.2734 | 4.3107 | -2.8849 |
| 1 | 4.4166 | -1.3813 | -1.4348 | 6.4773 | -0.3012 | -1.2156 | 0.7677 | 2.3526 | -1.2279 |
| 1 | 4.7409 | -3.3128 | -2.2751 | 8.0962 | 0.7426 | 0.0950 | 3.0887 | 3.2348 | -2.4340 |
| 1 | 4.7600 | -3.5257 | 0.6551 | 5.8721 | -1.5433 | 0.9202 | 2.8529 | 4.4694 | -0.5131 |
| 1 | 3.3395 | -0.9248 | 1.3349 | 3.5128 | 0.0987 | 1.4296 | 0.6271 | 3.1859 | 1.1506 |
| 1 | 4.7678 | -3.0102 | 3.0770 | 5.8559 | -0.6906 | 3.2530 | 1.2571 | 4.9179 | 2.8272 |
| 1 | 2.8096 | -3.8940 | 2.1064 | 4.6091 | -2.4629 | 2.3162 | 2.3080 | 6.3233 | 1.2519 |
| 1 | 3.7310 | -1.6858 | 3.6520 | 4.2817 | -0.0604 | 3.8038 | 2.6132 | 3.8120 | 2.5057 |
| 1 | 2.4911 | 5.1582 | 0.4795 | -0.3684 | 5.5747 | 0.6896 | -6.1838 | 2.4191 | 0.2390 |
| 1 | 4.6736 | 4.6881 | -0.5580 | 1.8203 | 5.7770 | -0.4675 | -5.1950 | 4.5221 | -0.6389 |
| 1 | 2.4959 | 3.9978 | -2.2303 | -0.0785 | 4.3799 | -2.0661 | -5.4575 | 2.4606 | -2.5261 |
| 1 | 3.4609 | 2.0312 | -1.3680 | 1.4071 | 2.8072 | -1.0287 | -3.0480 | 2.6996 | -1.8414 |
| 1 | 5.1702 | 2.8274 | -2.6733 | 2.6053 | 4.0990 | -2.6184 | -3.7031 | 4.7122 | -2.9299 |
| 1 | 5.6443 | 2.3610 | 0.7484 | 3.4792 | 4.2083 | 0.7328 | -2.7518 | 4.7673 | 0.3991 |
| 1 | 2.6701 | 1.5982 | 1.0434 | 1.1213 | 2.3432 | 1.3859 | -2.6482 | 1.6975 | 0.4193 |
| 1 | 4.8219 | 2.3465 | 3.1090 | 3.0073 | 3.9435 | 3.2031 | -2.6884 | 3.6314 | 2.7742 |
| 1 | 2.6424 | 2.9883 | 3.6014 | 0.7419 | 3.5880 | 3.8438 | -2.2040 | 1.5672 | 3.7932 |
| 1 | 4.1523 | 0.7159 | 2.8711 | 3.0309 | 2.1723 | 2.9895 | -1.2197 | 2.8535 | 2.0961 |
| 1 | -1.8692 | 3.2620 | -3.3185 | -3.4199 | 1.4871 | -2.9566 | -5.4105 | -2.9516 | -2.8516 |
| 1 | -3.3615 | 4.9139 | -0.4216 | -5.6315 | 2.8196 | -0.5092 | -4.5173 | -3.7279 | 0.5544 |
| 1 | -1.4999 | 5.2847 | -1.9712 | -3.9781 | 3.9292 | -2.0390 | -6.2992 | -2.5482 | -0.7414 |
| 1 | -0.5923 | 2.4799 | -1.1901 | -1.9956 | 1.8407 | -0.9784 | -3.9506 | -0.8427 | -1.6909 |
| 1 | 0.7310 | 2.6946 | -2.9032 | -1.4405 | 2.4715 | -3.0545 | -5.3355 | -0.1898 | -3.2060 |
| 1 | 0.6677 | 5.2100 | -0.6357 | -1.9854 | 4.8716 | -0.6294 | -6.4220 | 0.1090 | -0.1612 |
| 1 | -0.6622 | 3.1108 | 1.1807 | -2.6901 | 2.7927 | 1.5136 | -3.6208 | -0.1450 | 1.0011 |
| 1 | 1.1433 | 4.3389 | 2.3530 | -1.6651 | 4.8259 | 2.4188 | -5.3825 | 1.1901 | 2.3047 |
| 1 | -0.2217 | 6.6255 | 1.4113 | -3.6390 | 6.1714 | 0.9078 | -6.6727 | -1.3200 | 2.2604 |
| 1 | -0.4641 | 4.8472 | 2.9365 | -3.4049 | 4.7819 | 2.7938 | -4.7949 | -0.2483 | 3.1615 |
| 8 | -6.2250 | 0.4654 | -2.0251 | -5.7301 | -2.5421 | -1.5402 | 0.2879 | -3.5533 | -3.3307 |
| 8 | -5.1210 | 3.0891 | -1.9649 | -6.1716 | 0.2339 | -1.6991 | -2.2376 | -2.4239 | -2.7973 |
| 8 | -2.8088 | 2.9818 | 0.0562 | -4.1908 | 1.5487 | 0.2676 | -3.0209 | -2.4334 | -0.0543 |
| 8 | -5.4086 | 0.8931 | 1.4593 | -5.1929 | -1.5103 | 1.8747 | -0.4292 | -5.0342 | -0.1111 |
| 8 | -4.2091 | 1.4198 | 3.8244 | -4.1037 | -0.1807 | 4.0667 | -2.7413 | -4.0751 | 2.4943 |
| 8 | -3.0057 | -4.3184 | -2.5304 | -0.5888 | -4.8986 | -2.2355 | 5.4447 | -2.9701 | -2.7043 |
| 8 | -5.0414 | -2.3097 | -2.3360 | -3.0911 | -3.6288 | -2.1129 | 2.9933 | -4.3108 | -2.7818 |
| 8 | -4.5297 | -0.5531 | -0.1302 | -3.6720 | -2.2763 | 0.3038 | 1.0986 | -3.4009 | -0.7141 |
| 8 | -3.2061 | -3.7526 | 1.0327 | -0.9868 | -4.5292 | 1.3464 | 4.3273 | -2.6399 | 0.7252 |

| | | | | | | | | | |
|---|---|---|---|---|---|---|---|---|---|
| 8 | -5.2344 | -2.8466 | 2.6913 | -3.3332 | -4.4319 | 3.0001 | 2.8317 | -4.1346 | 2.4373 |
| 8 | 2.7134 | -3.1681 | -2.7346 | 3.9789 | -1.8022 | -2.9422 | 4.8364 | 3.5827 | -1.7069 |
| 8 | -0.1798 | -3.5148 | -2.9630 | 1.2693 | -2.8502 | -2.7324 | 5.7734 | 1.0568 | -2.4544 |
| 8 | -1.4788 | -3.4073 | -0.4654 | 0.4532 | -3.4662 | -0.1018 | 4.6768 | -1.0351 | -0.9126 |
| 8 | 1.8072 | -4.7592 | 0.4320 | 4.0766 | -3.4063 | 0.3487 | 4.6614 | 1.8125 | 1.4460 |
| 8 | -0.3976 | -6.1757 | 1.4274 | 2.7299 | -5.6689 | 1.3429 | 6.6695 | 0.2874 | 2.3094 |
| 8 | 6.8018 | -0.4556 | -1.5323 | 4.8936 | 1.4813 | -1.7139 | -0.4169 | 3.8563 | -2.9308 |
| 8 | 5.4860 | -3.1167 | -1.6866 | 7.4034 | 0.2749 | 0.5746 | 2.2093 | 2.8526 | -2.5805 |
| 8 | 2.9383 | -2.8281 | -0.0881 | 4.2711 | -1.2005 | -0.3513 | 2.9202 | 2.5022 | 0.1759 |
| 8 | 5.3702 | -0.9319 | 1.7817 | 5.0943 | 1.3805 | 1.8173 | 0.3925 | 5.1189 | 0.4063 |
| 8 | 2.7079 | -3.1979 | 2.7697 | 4.1770 | -1.9180 | 2.9886 | 2.8513 | 5.6604 | 1.6971 |
| 8 | 3.3303 | 4.4724 | -2.0722 | 0.5095 | 5.1376 | -1.8798 | -5.5619 | 3.3925 | -2.2883 |
| 8 | 5.4350 | 2.4654 | -1.8181 | 3.0592 | 3.7963 | -1.8206 | -3.0619 | 4.7443 | -2.2055 |
| 8 | 4.8345 | 0.5608 | 0.0746 | 3.5226 | 2.1686 | 0.3300 | -1.1822 | 3.6022 | -0.3247 |
| 8 | 3.2320 | 3.5592 | 1.4535 | 0.9499 | 4.4008 | 1.6798 | -4.3714 | 2.6668 | 1.0832 |
| 8 | 2.8367 | 2.0463 | 3.6792 | 1.3831 | 2.8767 | 3.9678 | -2.6258 | 1.5511 | 2.9269 |
| 8 | -2.4783 | 3.5755 | -2.6343 | -4.1761 | 1.9320 | -2.5376 | -4.8850 | -3.2475 | -2.0941 |
| 8 | 0.3156 | 3.5579 | -2.7243 | -1.4789 | 3.2294 | -2.4439 | -5.8381 | -0.6966 | -2.5619 |
| 8 | 1.6207 | 3.4114 | -0.2116 | -0.5931 | 3.5695 | 0.2060 | -4.7796 | 1.2585 | -0.7411 |
| 8 | -1.6465 | 4.8770 | 0.6459 | -4.2236 | 3.8220 | 0.5542 | -4.7935 | -1.8604 | 1.2418 |
| 8 | 0.5817 | 6.1932 | 1.7280 | -2.7785 | 6.1508 | 1.3457 | -6.7137 | -0.3579 | 2.3300 |
| 1 | -6.2076 | 1.1109 | -2.7448 | -6.0623 | -2.0184 | -2.2833 | -0.2937 | -3.1086 | -3.9619 |
| 6 | -1.9651 | 0.4443 | -2.4213 | 2.3977 | 0.9416 | -3.2375 | 1.9552 | 0.5306 | 3.7810 |
| 7 | -0.7794 | 0.5813 | -2.9581 | 1.2950 | 0.7625 | -3.9346 | 1.0967 | -0.3491 | 4.2417 |
| 6 | 0.1019 | 0.3029 | -1.9435 | 0.3515 | 0.4101 | -3.0082 | 0.1615 | -0.5030 | 3.2467 |
| 6 | -0.5782 | 0.0200 | -0.7662 | 0.8923 | 0.3935 | -1.7401 | 0.4870 | 0.2928 | 2.1610 |
| 7 | -1.8959 | 0.1237 | -1.0954 | 2.2080 | 0.7233 | -1.9092 | 1.6347 | 0.9421 | 2.5206 |
| 6 | 1.5445 | 0.5181 | -2.0912 | -1.0456 | 0.0015 | -3.3380 | -0.8808 | -1.5626 | 3.2395 |
| 8 | 1.9999 | 1.3134 | -2.9208 | -1.9026 | 0.8234 | -3.7116 | -0.6710 | -2.6883 | 3.7202 |
| 7 | 2.3253 | -0.2131 | -1.2626 | -1.2714 | -1.2981 | -3.1762 | -2.0132 | -1.2266 | 2.6119 |
| 7 | -0.0385 | -0.2376 | 0.4842 | 0.2063 | 0.1943 | -0.5535 | -0.1263 | 0.4037 | 0.9038 |
| 7 | -0.8981 | -0.1063 | 1.4184 | 0.9363 | -0.0864 | 0.4448 | 0.7148 | 0.0917 | -0.0002 |
| 7 | -0.4572 | -0.3207 | 2.6130 | 0.2541 | -0.2325 | 1.5559 | 0.2561 | 0.0522 | -1.2104 |
| 6 | -1.3582 | -0.0649 | 3.7191 | 0.9922 | -0.3502 | 2.8003 | 1.2113 | -0.2592 | -2.2558 |
| 6 | 0.9359 | -0.6203 | 2.8755 | -1.1395 | 0.1589 | 1.5832 | -1.1202 | 0.3805 | -1.5321 |
| 1 | -2.9114 | 0.5437 | -2.9444 | 3.3649 | 1.2234 | -3.6418 | 2.8127 | 0.9267 | 4.3154 |
| 1 | -2.6933 | -0.0044 | -0.4752 | 2.9014 | 0.8604 | -1.1723 | 2.1728 | 1.5534 | 1.9052 |
| 1 | 1.8875 | -0.7020 | -0.4854 | -0.4823 | -1.8988 | -2.9375 | -2.1501 | -0.2612 | 2.3312 |
| 1 | 3.2833 | 0.0850 | -1.1160 | -2.1911 | -1.7067 | -3.2996 | -2.6947 | -1.9359 | 2.3546 |
| 1 | -1.4350 | -0.9571 | 4.3543 | 1.0945 | 0.6291 | 3.2974 | 0.9376 | -1.2057 | -2.7482 |
| 1 | -0.9788 | 0.7680 | 4.3272 | 0.4498 | -1.0295 | 3.4693 | 1.2563 | 0.5596 | -2.9868 |
| 1 | -2.3463 | 0.2035 | 3.3330 | 1.9800 | -0.7817 | 2.6019 | 2.1950 | -0.3613 | -1.7802 |
| 1 | 1.0266 | -0.9030 | 3.9285 | -1.5457 | -0.0356 | 2.5836 | -1.2584 | 1.4746 | -1.5080 |
| 1 | 1.2762 | -1.4531 | 2.2489 | -1.2301 | 1.2309 | 1.3294 | -1.3414 | -0.0133 | -2.5300 |
| 1 | 1.5579 | 0.2700 | 2.6857 | -1.7032 | -0.4019 | 0.8237 | -1.7911 | -0.0934 | -0.7968 |
| **HP-β-CD:DTIC** | | | | | | | | | |

| Atomic Number | K03_03 | | | K04_03 | | | K04_01 | | |
|---|---|---|---|---|---|---|---|---|---|
| | x | y | z | x | y | z | x | y | z |
| 6 | 0.9135 | -4.8971 | -0.4853 | 4.6357 | -3.7648 | -0.9372 | -1.4455 | -4.8840 | 0.0601 |
| 6 | 0.2972 | -5.0733 | -1.8940 | 4.1218 | -4.1529 | -2.3283 | -2.3704 | -5.4199 | -1.0416 |
| 8 | 1.2194 | -4.5895 | -2.8658 | 4.5294 | -3.1692 | -3.2607 | -1.5865 | -5.9941 | -2.0644 |
| 6 | -1.0456 | -4.3708 | -2.0484 | 2.6032 | -4.2629 | -2.3382 | -3.2731 | -4.3272 | -1.5916 |
| 8 | -1.6536 | -4.8188 | -3.2312 | 2.2322 | -4.7718 | -3.5962 | -4.1434 | -4.9378 | -2.5153 |
| 6 | -1.9234 | -4.5856 | -0.8185 | 2.0888 | -5.1210 | -1.1918 | -3.9955 | -3.7006 | -0.4105 |
| 8 | -3.1026 | -3.8536 | -1.0891 | 0.6756 | -4.9524 | -1.2136 | -4.8960 | -2.6990 | -0.8518 |
| 6 | -1.1239 | -4.0510 | 0.3711 | 2.7042 | -4.6178 | 0.1153 | -2.9531 | -3.1108 | 0.5368 |
| 8 | -0.0333 | -4.9599 | 0.5415 | 4.1218 | -4.7015 | -0.0086 | -2.1965 | -4.2112 | 1.0502 |
| 6 | -1.8543 | -3.9209 | 1.6988 | 2.2874 | -5.3810 | 1.3697 | -3.5939 | -2.3497 | 1.6988 |
| 8 | -1.2273 | -2.9791 | 2.5452 | 1.0245 | -4.9300 | 1.8278 | -3.6856 | -0.9517 | 1.4401 |
| 6 | -4.3627 | -4.4229 | -0.8601 | -0.1649 | -6.0584 | -1.0520 | -6.2322 | -3.1305 | -1.0404 |
| 6 | -5.2176 | -4.0856 | -2.0850 | -1.0941 | -6.1482 | -2.2654 | -6.7586 | -2.4804 | -2.3061 |
| 8 | -4.6043 | -4.6519 | -3.2267 | -0.2837 | -6.2173 | -3.4223 | -6.0888 | -3.0533 | -3.4141 |
| 6 | -5.2746 | -2.5745 | -2.2023 | -2.0235 | -4.9441 | -2.3181 | -6.4879 | -0.9886 | -2.2228 |
| 8 | -6.1360 | -2.2219 | -3.2595 | -2.9445 | -5.1576 | -3.3605 | -7.3087 | -0.3566 | -3.1804 |
| 6 | -5.7139 | -1.9288 | -0.8937 | -2.7225 | -4.7772 | -0.9792 | -6.6533 | -0.3904 | -0.8061 |
| 8 | -5.4581 | -0.5435 | -1.0912 | -3.4918 | -3.5954 | -1.0753 | -5.4831 | 0.3938 | -0.5153 |
| 6 | -4.9031 | -2.4510 | 0.2903 | -1.6839 | -4.6826 | 0.1341 | -6.8558 | -1.3715 | 0.3817 |
| 8 | -4.9663 | -3.8819 | 0.2941 | -0.9349 | -5.9133 | 0.1297 | -7.0412 | -2.7379 | 0.0301 |
| 6 | -5.3083 | -1.9126 | 1.6476 | -2.2970 | -4.4500 | 1.4998 | -8.0660 | -0.9678 | 1.2229 |
| 8 | -6.7092 | -1.8034 | 1.7995 | -3.4676 | -5.2125 | 1.6530 | -7.9670 | 0.3785 | 1.6328 |
| 6 | -7.0572 | -1.5401 | 3.1523 | -4.1028 | -4.9218 | 2.8813 | -6.9718 | 0.6145 | 2.6191 |
| 6 | -8.3207 | -0.6951 | 3.1837 | -5.5145 | -5.4868 | 2.8764 | -7.0960 | 2.0791 | 3.0353 |
| 6 | -8.7683 | -0.4140 | 4.6069 | -6.1793 | -5.2740 | 4.2331 | -8.3606 | 2.3426 | 3.8383 |
| 8 | -8.0706 | 0.5489 | 2.5440 | -6.2871 | -4.9097 | 1.8342 | -5.9274 | 2.4915 | 3.7346 |
| 6 | -6.5035 | 0.3520 | -0.8660 | -4.7805 | -3.6175 | -0.5242 | -5.7151 | 1.7686 | -0.2811 |
| 6 | -6.4202 | 1.4438 | -1.9288 | -5.7493 | -3.1230 | -1.6000 | -5.3295 | 2.6228 | -1.5008 |
| 8 | -6.4463 | 0.8068 | -3.1905 | -5.5659 | -3.9269 | -2.7503 | -5.7912 | 1.9816 | -2.6769 |
| 6 | -5.1544 | 2.2656 | -1.7336 | -5.4964 | -1.6569 | -1.9031 | -3.8331 | 2.8953 | -1.6044 |
| 8 | -5.1948 | 3.3504 | -2.6315 | -6.5034 | -1.2191 | -2.7843 | -3.6436 | 3.8158 | -2.6508 |
| 6 | -5.0410 | 2.7395 | -0.2904 | -5.5067 | -0.8731 | -0.6008 | -3.3311 | 3.3865 | -0.2608 |
| 8 | -3.7578 | 3.3334 | -0.1988 | -5.2533 | 0.4803 | -0.9362 | -1.9510 | 3.6912 | -0.3757 |
| 6 | -5.1683 | 1.5670 | 0.6799 | -4.4643 | -1.4370 | 0.3621 | -3.6128 | 2.2842 | 0.7566 |
| 8 | -6.4142 | 0.9122 | 0.4173 | -4.8457 | -2.7960 | 0.6246 | -5.0341 | 2.1800 | 0.8644 |
| 6 | -5.1944 | 1.9772 | 2.1459 | -4.3835 | -0.6647 | 1.6730 | -3.0887 | 2.5938 | 2.1485 |
| 8 | -6.2768 | 2.8329 | 2.4346 | -5.6548 | -0.3370 | 2.1941 | -3.6586 | 3.7780 | 2.6549 |
| 6 | -3.6156 | 4.4400 | 0.6547 | -6.1653 | 1.4372 | -0.4851 | -1.5458 | 4.8811 | 0.2716 |
| 6 | -3.2864 | 5.6593 | -0.2053 | -6.4110 | 2.4220 | -1.6255 | -1.0198 | 5.8612 | -0.7779 |
| 8 | -4.3041 | 5.7947 | -1.1800 | -6.9003 | 1.6994 | -2.7377 | -1.9985 | 6.0142 | -1.7877 |
| 6 | -1.9390 | 5.4634 | -0.8795 | -5.1078 | 3.1198 | -1.9626 | 0.2825 | 5.3553 | -1.3720 |
| 8 | -1.6385 | 6.6693 | -1.5535 | -5.3744 | 4.0623 | -2.9729 | 0.7330 | 6.3418 | -2.2702 |
| 6 | -0.8688 | 5.0728 | 0.1327 | -4.5118 | 3.7637 | -0.7182 | 1.2827 | 5.0627 | -0.2628 |
| 8 | 0.3040 | 4.7022 | -0.5865 | -3.2085 | 4.1900 | -1.0916 | 2.4245 | 4.4532 | -0.8490 |

| | | | | | | | | | |
|---|---|---|---|---|---|---|---|---|---|
| 6 | -1.3328 | 3.8828 | 0.9737 | -4.3999 | 2.7505 | 0.4244 | 0.6562 | 4.0874 | 0.7361 |
| 8 | -2.5897 | 4.2032 | 1.5779 | -5.6666 | 2.1157 | 0.6463 | -0.5664 | 4.6314 | 1.2351 |
| 6 | -0.3721 | 3.5456 | 2.0971 | -3.9883 | 3.3811 | 1.7394 | 1.5375 | 3.8442 | 1.9453 |
| 8 | -0.2624 | 4.6448 | 2.9845 | -4.9787 | 4.2983 | 2.1704 | 1.7557 | 5.0644 | 2.6278 |
| 6 | 1.0612 | 5.0242 | 3.2977 | -4.4959 | 5.6025 | 2.4237 | 3.1068 | 5.3029 | 2.9860 |
| 6 | 1.6409 | 4.2897 | 4.5142 | -3.8271 | 5.7949 | 3.8068 | 3.5241 | 4.6952 | 4.3375 |
| 6 | 1.3162 | 2.8018 | 4.5458 | -3.5402 | 4.4982 | 4.5531 | 2.9547 | 3.2998 | 4.5881 |
| 8 | 3.0534 | 4.5117 | 4.4118 | -2.6164 | 6.5184 | 3.5411 | 4.9416 | 4.7117 | 4.4067 |
| 6 | 1.2664 | 5.7151 | -0.7376 | -2.8650 | 5.5377 | -0.9701 | 3.6003 | 5.2031 | -0.9853 |
| 6 | 1.7524 | 5.6798 | -2.1980 | -2.1877 | 5.9737 | -2.2708 | 4.1327 | 5.0054 | -2.4077 |
| 8 | 0.6350 | 5.7255 | -3.0725 | -3.0667 | 5.7326 | -3.3507 | 3.1905 | 5.4921 | -3.3425 |
| 6 | 2.6063 | 4.4446 | -2.4379 | -0.8840 | 5.2113 | -2.4487 | 4.3673 | 3.5222 | -2.6239 |
| 8 | 3.1936 | 4.4961 | -3.7125 | -0.2678 | 5.7054 | -3.6121 | 4.8787 | 3.3411 | -3.9222 |
| 6 | 3.6729 | 4.3018 | -1.3544 | -0.0107 | 5.3955 | -1.2145 | 5.2905 | 2.9520 | -1.5557 |
| 8 | 4.2653 | 3.0457 | -1.6362 | 1.1566 | 4.6109 | -1.4127 | 5.1542 | 1.5382 | -1.6686 |
| 6 | 3.0145 | 4.3059 | 0.0240 | -0.7870 | 4.9599 | 0.0292 | 4.8351 | 3.3680 | -0.1515 |
| 8 | 2.3184 | 5.5475 | 0.1737 | -1.9820 | 5.7418 | 0.1089 | 4.5930 | 4.7725 | -0.0794 |
| 6 | 3.9719 | 4.1505 | 1.2076 | -0.0386 | 5.1800 | 1.3321 | 5.8441 | 2.9773 | 0.9160 |
| 8 | 3.5307 | 3.1017 | 2.0577 | -0.7876 | 4.7137 | 2.4353 | 5.2627 | 2.9259 | 2.2045 |
| 6 | 5.6534 | 2.8877 | -1.6348 | 2.3960 | 5.2696 | -1.3682 | 6.2721 | 0.7071 | -1.8581 |
| 6 | 5.9510 | 1.8638 | -2.7294 | 3.2443 | 4.7798 | -2.5466 | 5.9464 | -0.2219 | -3.0271 |
| 8 | 5.4610 | 2.3874 | -3.9472 | 2.5153 | 4.9949 | -3.7383 | 5.7055 | 0.5682 | -4.1770 |
| 6 | 5.2697 | 0.5452 | -2.3763 | 3.5975 | 3.3134 | -2.3637 | 4.7279 | -1.0649 | -2.6809 |
| 8 | 5.6789 | -0.4333 | -3.3060 | 4.4528 | 2.9162 | -3.4101 | 4.5319 | -1.9869 | -3.7284 |
| 6 | 5.5981 | 0.1346 | -0.9460 | 4.2450 | 3.1262 | -1.0049 | 4.9240 | -1.7591 | -1.3424 |
| 8 | 4.7283 | -0.9488 | -0.6446 | 4.4894 | 1.7384 | -0.8858 | 3.6595 | -2.3149 | -1.0050 |
| 6 | 5.3668 | 1.2794 | 0.0415 | 3.3077 | 3.6448 | 0.0889 | 5.3753 | -0.7694 | -0.2636 |
| 8 | 6.1248 | 2.4066 | -0.3937 | 3.0559 | 5.0318 | -0.1502 | 6.5366 | -0.0590 | -0.7098 |
| 6 | 5.7223 | 0.9151 | 1.4676 | 3.8971 | 3.4848 | 1.4747 | 5.6592 | -1.4453 | 1.0594 |
| 8 | 6.9755 | 0.2605 | 1.4903 | 5.2133 | 3.9894 | 1.4707 | 6.5067 | -2.5551 | 0.8613 |
| 6 | 5.2642 | -2.0280 | 0.0882 | 5.6632 | 1.3397 | -0.2131 | 3.6770 | -3.5732 | -0.3732 |
| 6 | 5.2642 | -3.2588 | -0.8108 | 6.4705 | 0.4632 | -1.1637 | 3.0938 | -4.6131 | -1.3275 |
| 8 | 6.0478 | -2.9717 | -1.9551 | 6.7133 | 1.2024 | -2.3477 | 3.8036 | -4.5397 | -2.5508 |
| 6 | 3.8399 | -3.5831 | -1.2215 | 5.7048 | -0.8076 | -1.4976 | 1.6109 | -4.3746 | -1.5595 |
| 8 | 3.9031 | -4.7791 | -1.9725 | 6.5799 | -1.6289 | -2.2473 | 1.1588 | -5.4262 | -2.3759 |
| 6 | 2.9232 | -3.6791 | -0.0078 | 5.1699 | -1.4988 | -0.2437 | 0.8837 | -4.3130 | -0.2214 |
| 8 | 1.5729 | -3.6533 | -0.4719 | 4.1951 | -2.4730 | -0.6229 | -0.4854 | -4.0298 | -0.5036 |
| 6 | 3.0885 | -2.4773 | 0.9195 | 4.4531 | -0.4879 | 0.6586 | 1.5423 | -3.2049 | 0.6224 |
| 8 | 4.4770 | -2.2769 | 1.2252 | 5.3360 | 0.6014 | 0.9358 | 2.9220 | -3.5280 | 0.8114 |
| 6 | 2.3270 | -2.6606 | 2.2167 | 4.0280 | -1.0619 | 1.9990 | 0.9530 | -2.8995 | 1.9995 |
| 8 | 2.7759 | -3.8377 | 2.8510 | 5.1073 | -1.7035 | 2.6473 | 1.2994 | -3.7900 | 3.0381 |
| 6 | 1.7562 | -4.6916 | 3.3365 | 4.9612 | -3.1087 | 2.7245 | 0.6145 | -5.0264 | 3.0290 |
| 6 | 2.2449 | -6.1265 | 3.2586 | 6.3147 | -3.7913 | 2.7781 | 1.5245 | -6.1604 | 2.5415 |
| 6 | 1.2228 | -7.0779 | 3.8694 | 6.1250 | -5.2899 | 2.9876 | 2.3131 | -6.7903 | 3.6804 |
| 8 | 2.4483 | -6.4133 | 1.8787 | 6.9482 | -3.5358 | 1.5298 | 0.6664 | -7.1003 | 1.9033 |
| 1 | 1.6294 | -5.7046 | -0.2766 | 5.7317 | -3.8300 | -0.8661 | -0.9553 | -5.7293 | 0.5703 |

| | | | | | | | | | |
|---|---|---|---|---|---|---|---|---|---|
| 1 | 0.1498 | -6.1436 | -2.0944 | 4.5463 | -5.1401 | -2.5830 | -3.0192 | -6.1868 | -0.5794 |
| 1 | 1.3681 | -3.6647 | -2.6094 | 4.1298 | -3.3900 | -4.1117 | -2.1814 | -6.1499 | -2.8107 |
| 1 | -0.8613 | -3.2713 | -2.0854 | 2.1822 | -3.2500 | -2.1768 | -2.6465 | -3.5536 | -2.0790 |
| 1 | -2.5870 | -4.5437 | -3.2107 | 1.2954 | -5.0314 | -3.5568 | -4.6403 | -4.2489 | -2.9881 |
| 1 | -2.1529 | -5.6555 | -0.6672 | 2.3573 | -6.1807 | -1.3527 | -4.5487 | -4.4772 | 0.1501 |
| 1 | 3.0561 | -3.1805 | -4.3569 | 2.4114 | -3.5571 | 0.2510 | -2.2793 | -2.4146 | -0.0005 |
| 1 | -1.8344 | -4.8966 | 2.2079 | 3.0281 | -5.1810 | 2.1577 | -2.9603 | -2.4640 | 2.5893 |
| 1 | -2.9098 | -3.6639 | 1.5120 | 2.2871 | -6.4689 | 1.1884 | -4.5886 | -2.7678 | 1.9339 |
| 1 | -1.4621 | -2.0972 | 2.2109 | 0.3369 | -5.4493 | 1.3834 | -4.1561 | -0.7760 | 0.6075 |
| 1 | -4.2871 | -5.5098 | -0.7037 | 0.4161 | -6.9862 | -0.9337 | -6.2775 | -4.2279 | -1.1060 |
| 1 | -6.2322 | -4.4932 | -1.9330 | -1.7085 | -7.0599 | -2.1612 | -7.8441 | -2.6683 | -2.3590 |
| 1 | -5.0200 | -4.2463 | -3.9991 | -0.8747 | -6.1732 | -4.1854 | -6.2781 | -2.5121 | -4.1904 |
| 1 | -4.2416 | -2.2341 | -2.4116 | -1.4100 | -4.0390 | -2.5012 | -5.4275 | -0.8561 | -2.4779 |
| 1 | -6.1112 | -1.2539 | -3.3480 | -3.6947 | -4.5515 | -3.2284 | -6.9916 | 0.5569 | -3.2397 |
| 1 | -6.7895 | -2.1062 | -0.7255 | -3.3718 | -5.6490 | -0.7889 | -7.5288 | 0.2784 | -0.8524 |
| 1 | -3.8518 | -2.1504 | 0.1623 | -0.9932 | -3.8468 | -0.0726 | -5.9586 | -1.2920 | 1.0103 |
| 1 | -4.8334 | -0.9244 | 1.7795 | -2.5319 | -3.3694 | 1.5906 | -3.4285 | -1.1037 | -1.8200 |
| 1 | -4.8814 | -2.5861 | 2.4134 | -1.5580 | -4.6954 | 2.2859 | -8.9803 | -1.0544 | 0.6183 |
| 1 | -7.4735 | -0.1666 | -0.9132 | -5.0474 | -4.6254 | -0.1789 | -6.7840 | 1.9087 | -0.0525 |
| 1 | -7.2912 | 2.1111 | -1.8020 | -6.7790 | -3.2249 | -1.2115 | -5.8252 | 3.6032 | -1.3786 |
| 1 | -6.2986 | 1.4829 | -3.8633 | -6.0754 | -3.5178 | -3.4619 | -5.5197 | 2.5383 | -3.4189 |
| 1 | -4.2799 | 1.6125 | -1.9360 | -4.4913 | -1.5606 | -2.3602 | -3.3153 | 1.9415 | -1.8214 |
| 1 | -4.6795 | 4.0796 | -2.2461 | -6.4369 | -0.2522 | -2.8685 | -2.9080 | 4.4071 | -2.4128 |
| 1 | -5.8339 | 3.4797 | -0.0781 | -6.4953 | -0.9680 | -0.1180 | -3.8964 | 4.2852 | 0.0436 |
| 1 | -4.3255 | 0.8686 | 0.5140 | -3.4630 | -1.4346 | -0.1050 | -3.1764 | 1.3308 | 0.3977 |
| 1 | -4.2541 | 2.4973 | 2.3842 | -3.7784 | 0.2416 | 1.4964 | -1.9965 | 2.7284 | 2.0886 |
| 1 | -5.2350 | 1.0633 | 2.7645 | -3.8319 | -1.2765 | 2.4084 | -3.3004 | 1.7275 | 2.8014 |
| 1 | -7.0454 | 2.2626 | 2.5908 | -5.8215 | 0.5865 | 1.9598 | -4.5474 | 3.5317 | 2.9596 |
| 1 | -4.5371 | 4.5990 | 1.2393 | -7.1073 | 0.9629 | -0.1673 | -2.3971 | 5.3108 | 0.8214 |
| 1 | -3.2393 | 6.5507 | 0.4450 | -7.1486 | 3.1714 | -1.2862 | -0.8248 | 6.8266 | -0.2765 |
| 1 | -4.0019 | 6.4778 | -1.7942 | -6.7958 | 2.2736 | -3.5082 | -1.5627 | 6.4826 | -2.5135 |
| 1 | -2.0410 | 4.6238 | -1.5949 | -4.3912 | 2.3515 | -2.3125 | 0.0764 | 4.4000 | -1.8970 |
| 1 | -0.9356 | 6.4960 | -2.2005 | -4.5385 | 4.5050 | -3.2012 | 1.4936 | 5.9868 | -2.7617 |
| 1 | -0.6537 | 5.9165 | 0.8131 | -5.1321 | 4.6206 | -0.3976 | 1.5615 | 5.9911 | 0.2668 |
| 1 | -1.4357 | 2.9921 | 0.3271 | -3.6409 | 1.9978 | 0.1421 | 0.4617 | 3.1235 | 0.2246 |
| 1 | 0.6089 | 3.2883 | 1.6593 | -3.0206 | 3.8911 | 1.5927 | 2.4949 | 3.4070 | 1.6109 |
| 1 | -0.7412 | 2.6520 | 2.6238 | -3.8421 | 2.5887 | 2.4953 | 1.0466 | 3.1076 | 2.6032 |
| 1 | 0.8235 | 6.6988 | -0.5183 | -3.7547 | 6.1544 | -0.7626 | 3.4171 | 6.2676 | -0.7711 |
| 1 | 2.3516 | 6.5802 | -2.3957 | -1.9555 | 7.0511 | -2.1913 | 5.0882 | 5.5522 | -2.4959 |
| 1 | 0.2443 | 4.8408 | -3.0733 | -2.5402 | 5.7929 | -4.1591 | 3.3916 | 5.0725 | -4.1899 |
| 1 | 1.9484 | 3.5527 | -2.3368 | -1.1181 | 4.1314 | -2.5429 | 3.3874 | 3.0173 | -2.5096 |
| 1 | 3.7938 | 3.7336 | -3.7801 | 0.6012 | 5.2782 | -3.7068 | 5.0093 | 2.3867 | -4.0620 |
| 1 | 4.4175 | 5.1144 | -1.4357 | 0.2577 | 6.4635 | -1.1146 | 6.3330 | 3.2614 | -1.7508 |
| 1 | 2.2961 | 3.4633 | 0.0583 | -1.0566 | 3.8884 | -0.0605 | 3.8929 | 2.8385 | 0.0571 |
| 1 | 3.9989 | 5.1084 | 1.7517 | 0.2029 | 6.2516 | 1.4371 | 6.6570 | 3.7166 | 0.9410 |
| 1 | 4.9910 | 3.9334 | 0.8503 | 0.9114 | 4.6264 | 1.2944 | 6.2758 | 1.9969 | 0.6443 |

| | | | | | | | | | |
|---|---|---|---|---|---|---|---|---|---|
| 1 | 6.1663 | 3.8442 | -1.8202 | 2.2540 | 6.3593 | -1.4319 | 7.1717 | 1.3084 | -2.0596 |
| 1 | 7.0419 | 1.7002 | -2.7724 | 4.1814 | 5.3650 | -2.5520 | 6.8096 | -0.8917 | -3.1868 |
| 1 | 5.5364 | 1.6953 | -4.6159 | 2.9909 | 4.5528 | -4.4529 | 5.3374 | -0.0168 | -4.8518 |
| 1 | 4.1736 | 0.7075 | -2.4519 | 2.6557 | 2.7267 | -2.3780 | 3.8549 | -0.3883 | -2.5847 |
| 1 | 5.6782 | -1.3021 | -2.8650 | 5.0330 | 2.2083 | -3.0775 | 4.0558 | -2.7547 | -3.3674 |
| 1 | 6.6528 | -0.1862 | -0.8942 | 5.1928 | 3.6907 | -0.9621 | 5.6795 | -2.5565 | -1.4546 |
| 1 | 4.2945 | 1.5492 | 0.0442 | 2.3570 | 3.0805 | 0.0491 | 4.5687 | -0.0520 | -0.0659 |
| 1 | 4.9201 | 0.2690 | 1.8623 | 3.9003 | 2.4091 | 1.7274 | 4.6885 | -1.7713 | 1.4785 |
| 1 | 5.7376 | 1.8335 | 2.0794 | 3.2668 | 4.0132 | 2.2138 | 6.1022 | -0.7147 | 1.7605 |
| 1 | 6.2733 | -1.7780 | 0.4485 | 6.2421 | 2.2134 | 0.1242 | 4.7026 | -3.8506 | -0.0837 |
| 1 | 5.6882 | -4.1082 | -0.2475 | 7.4188 | 0.1953 | -0.6643 | 3.2173 | -5.6107 | -0.8660 |
| 1 | 5.8905 | -3.6943 | -2.5791 | 7.0695 | 0.5709 | -2.9877 | 3.3422 | -5.1217 | -3.1698 |
| 1 | 3.4770 | -2.7370 | -1.8418 | 4.8389 | -0.5119 | -2.1149 | 1.4876 | -3.3924 | -2.0607 |
| 1 | 3.0463 | -4.9197 | -2.4070 | 6.0308 | -2.1917 | -2.8155 | 0.1863 | -5.4161 | -2.3890 |
| 1 | 3.1162 | -4.6037 | 0.5632 | 5.9897 | -1.9753 | 0.3200 | 0.9623 | -5.2974 | 0.2701 |
| 1 | 2.6987 | -1.5884 | 0.4083 | 3.5506 | -0.1127 | 0.1422 | 1.4547 | -2.2622 | 0.0437 |
| 1 | 1.2489 | -2.7140 | 1.9762 | 3.1801 | -1.7520 | 1.8410 | -0.1467 | -2.8122 | 1.8938 |
| 1 | 2.4889 | -1.7795 | 2.8639 | 3.6699 | -0.2331 | 2.6301 | 1.3449 | -1.9230 | 2.3134 |
| 1 | 1.7227 | 4.8462 | 2.4340 | -3.7527 | 5.8938 | 1.6605 | 3.7798 | 4.9116 | 2.2036 |
| 1 | 1.0574 | 6.1060 | 3.4926 | -5.3607 | 6.2721 | 2.3288 | 3.2403 | 6.3940 | 3.0243 |
| 1 | 1.2667 | 4.7590 | 5.4396 | -4.4759 | 6.4285 | 4.4340 | 3.1762 | 5.3528 | 5.1508 |
| 1 | 1.8377 | 2.3182 | 5.3822 | -3.1590 | 4.7208 | 5.5584 | 3.3804 | 2.8977 | 5.5161 |
| 1 | 0.2390 | 2.6485 | 4.6854 | -4.4568 | 3.9039 | 4.6562 | 1.8631 | 3.3392 | 4.6991 |
| 1 | 1.6229 | 2.3082 | 3.6113 | -2.7910 | 3.8824 | 4.0347 | 3.1799 | 2.5977 | 3.7710 |
| 1 | 3.4705 | 4.2488 | 5.2401 | -2.2248 | 6.7798 | 4.3831 | 5.2471 | 4.1243 | 3.6956 |
| 1 | -7.1916 | -2.4866 | 3.7045 | -3.5224 | -5.3366 | 3.7257 | -7.1324 | -0.0544 | 3.4876 |
| 1 | -6.2503 | -0.9679 | 3.6483 | -4.1558 | -3.8221 | 3.0209 | -5.9555 | 0.4455 | 2.2247 |
| 1 | -9.1161 | -1.2368 | 2.6380 | -5.4593 | -6.5646 | 2.6537 | -7.1053 | 2.6821 | 2.1153 |
| 1 | -9.6520 | 0.2347 | 4.6031 | -7.2089 | -5.6491 | 4.2070 | -8.4660 | 3.4097 | 4.0671 |
| 1 | -9.0216 | -1.3431 | 5.1316 | -5.6386 | -5.7965 | 5.0329 | -9.2393 | 2.0126 | 3.2703 |
| 1 | -7.9686 | 0.0970 | 5.1604 | -6.2114 | -4.2030 | 4.4801 | -8.3390 | 1.7820 | 4.7850 |
| 1 | -7.6047 | 0.3634 | 1.7129 | -6.0561 | -3.9713 | 1.7739 | -5.9292 | 2.1017 | 4.6164 |
| 1 | 1.5007 | -4.4431 | 4.3825 | 4.3687 | -3.3888 | 3.6157 | 0.2738 | -5.2350 | 4.0566 |
| 1 | 0.8460 | -4.5947 | 2.7205 | 4.4470 | -3.4856 | 1.8264 | -0.2862 | -4.9659 | 2.3972 |
| 1 | 3.2054 | -6.2007 | 3.8015 | 6.9099 | -3.3561 | 3.6018 | 2.2343 | -5.7204 | 1.8160 |
| 1 | 1.5589 | -8.1193 | 3.7826 | 7.0918 | -5.8100 | 2.9881 | 3.0041 | -7.5602 | 3.3121 |
| 1 | 1.0625 | -6.8618 | 4.9334 | 5.6257 | -5.5029 | 3.9417 | 2.9038 | -6.0219 | 4.1969 |
| 1 | 0.2635 | -6.9816 | 3.3420 | 5.5153 | -5.6941 | 2.1661 | 1.6286 | -7.2511 | 4.4058 |
| 1 | 2.8607 | -7.2811 | 1.8101 | 7.8261 | -3.9319 | 1.5487 | 1.1982 | -7.8462 | 1.6040 |
| 1 | 3.5219 | 3.4439 | 2.9672 | -1.4358 | 5.3945 | 2.6946 | 4.6053 | 2.2094 | 2.1799 |
| 6 | 7.4908 | 0.0241 | 2.7905 | 5.9570 | 3.5681 | 2.5952 | 6.4819 | -3.4580 | 1.9457 |
| 6 | 6.9997 | -1.2852 | 3.4061 | 7.3996 | 3.9984 | 2.4093 | 7.1768 | -4.7398 | 1.5255 |
| 6 | 7.7010 | -1.5405 | 4.7334 | 8.2239 | 3.7090 | 3.6583 | 7.3202 | -5.6987 | 2.7015 |
| 8 | 5.5994 | -1.2424 | 3.6478 | 7.8893 | 3.2719 | 1.2893 | 6.3740 | -5.3037 | 0.4958 |
| 1 | 8.5864 | -0.0047 | 2.6932 | 5.5459 | 4.0041 | 3.5240 | 6.9756 | -3.0246 | 2.8343 |
| 1 | 7.2268 | 0.8607 | 3.4637 | 5.9182 | 2.4641 | 2.6810 | 5.4326 | -3.6957 | 2.2169 |

| | | | | | | | | | |
|---|---|---|---|---|---|---|---|---|---|
| 1 | 7.2356 | -2.1038 | 2.6999 | 7.4086 | 5.0823 | 2.1921 | 8.1736 | -4.4749 | 1.1281 |
| 1 | 7.3503 | -2.4833 | 5.1687 | 9.2755 | 3.9786 | 3.4968 | 7.7854 | -6.6387 | 2.3781 |
| 1 | 8.7899 | -1.5940 | 4.6096 | 7.8563 | 4.2820 | 4.5190 | 7.9457 | -5.2669 | 3.4931 |
| 1 | 7.4708 | -0.7308 | 5.4400 | 8.1784 | 2.6389 | 3.9020 | 6.3323 | -5.9322 | 3.1212 |
| 1 | 5.1459 | -1.6369 | 2.8897 | 8.7734 | 3.5934 | 1.0825 | 6.8370 | -6.0659 | 0.1323 |
| 6 | 1.5866 | 0.7216 | 0.7814 | -0.2928 | -2.4359 | 3.1262 | -0.3830 | 0.0771 | 3.0403 |
| 7 | 0.3510 | 0.4729 | 1.1486 | -0.9927 | -1.4330 | 3.5956 | 0.8697 | 0.4343 | 2.8848 |
| 6 | -0.2321 | -0.1658 | 0.0792 | -0.9237 | -0.4560 | 2.6306 | 1.0339 | 0.5906 | 1.5292 |
| 6 | 0.6918 | -0.3203 | -0.9433 | -0.1610 | -0.9030 | 1.5569 | -0.1642 | 0.3309 | 0.8747 |
| 7 | 1.8345 | 0.2710 | -0.4841 | 0.2253 | -2.1716 | 1.8927 | -1.0516 | -0.0053 | 1.8539 |
| 6 | -1.6761 | -0.4997 | 0.0828 | -1.4919 | 0.8988 | 2.8624 | 2.3469 | 0.8813 | 0.9213 |
| 8 | -2.2985 | -0.6807 | 1.1387 | -2.1513 | 1.1589 | 3.8676 | 3.3747 | 0.9696 | 1.6111 |
| 7 | -2.2665 | -0.5558 | -1.1247 | -1.2280 | 1.8223 | 1.9037 | 2.3540 | 1.0252 | -0.4180 |
| 7 | 0.5361 | -0.9449 | -2.1728 | 0.1240 | -0.1888 | 0.4087 | -0.4279 | 0.4834 | -0.4751 |
| 7 | 1.6588 | -1.1625 | -2.7284 | 1.0084 | -0.6861 | -0.3556 | -1.5001 | -0.0807 | -0.8597 |
| 7 | 1.6071 | -1.7052 | -3.9100 | 1.2160 | 0.0288 | -1.4260 | -1.8022 | 0.1423 | -2.1010 |
| 6 | 2.8774 | -2.1010 | -4.4935 | 2.2060 | -0.4270 | -2.3786 | -2.9658 | -0.5322 | -2.6334 |
| 6 | 0.3479 | -1.9681 | -4.5860 | 0.4129 | 1.2011 | -1.7133 | -1.0320 | 1.0679 | -2.9070 |
| 1 | 2.3360 | 1.2566 | 1.3669 | -0.1079 | -3.3858 | 3.6179 | -0.8685 | -0.1419 | 3.9856 |
| 1 | 2.7366 | 0.2087 | -0.9460 | 0.7534 | -2.8488 | 1.3507 | -2.0091 | -0.3493 | 1.7036 |
| 1 | -1.7325 | -0.4071 | -1.9707 | -0.5918 | 1.5711 | 1.1553 | 1.4924 | 0.9328 | -0.9455 |
| 1 | -3.2742 | -0.6476 | -1.1839 | -1.2940 | 2.8025 | 2.1702 | 3.2309 | 1.1726 | -0.9056 |
| 1 | -0.7272 | -3.0478 | 0.1082 | 1.7269 | -0.7703 | -3.3070 | -3.6913 | 0.2041 | -3.0128 |
| 1 | 2.8722 | -1.8781 | -5.5661 | 2.8927 | 0.3999 | -2.6164 | -2.6815 | -1.2118 | -3.4487 |
| 1 | 3.6790 | -1.5301 | -4.0099 | 2.7754 | -1.2472 | -1.9271 | -8.1564 | -1.6454 | 2.0898 |
| 1 | -0.1739 | -2.8407 | -4.1630 | 0.6379 | 1.5264 | -2.7337 | -1.5630 | 1.2212 | -3.8506 |
| 1 | -0.3005 | -1.0885 | -4.4919 | -0.6540 | 0.9548 | -1.6223 | -0.9371 | 2.0208 | -2.3603 |
| 1 | 0.5680 | -2.1615 | -5.6392 | 0.6325 | 2.0263 | -1.0148 | -0.0240 | 0.6770 | -3.1049 |
| **HE-β-CD:DTIC** | | | | | | | | | |
| **Atomic Number** | **K04_01** | | | **K03_02** | | | **K03_03** | | |
| | **x** | **y** | **z** | **x** | **y** | **z** | **x** | **y** | **z** |
| 6 | -1.0737 | -5.7276 | -0.3264 | 1.2772 | 5.2684 | -0.3508 | -0.4645 | 5.7005 | -0.7967 |
| 6 | -1.7139 | -5.8597 | -1.7055 | 0.8487 | 5.6550 | 1.0762 | 0.1130 | 5.4220 | -2.1895 |
| 8 | -0.7148 | -6.0231 | -2.6895 | 1.9532 | 5.5692 | 1.9622 | -0.9088 | 4.8700 | -3.0103 |
| 6 | -2.4982 | -4.5924 | -1.9881 | -0.3031 | 4.7766 | 1.5376 | 1.2720 | 4.4439 | -2.0637 |
| 8 | -3.0771 | -4.7488 | -3.2683 | -0.7700 | 5.2409 | 2.7767 | 1.8969 | 4.2964 | -3.3107 |
| 6 | -3.5111 | -4.3020 | -0.8892 | -1.4034 | 4.8147 | 0.4863 | 2.2545 | 4.8727 | -0.9819 |
| 8 | -3.9837 | -2.9610 | -1.0522 | -2.4296 | 3.9518 | 0.9416 | 3.1276 | 3.7562 | -0.8392 |
| 6 | -2.8581 | -4.3969 | 0.4992 | -0.8832 | 4.3903 | -0.8869 | 1.5412 | 5.1650 | 0.3412 |
| 8 | -2.1095 | -5.6042 | 0.6207 | 0.1895 | 5.2692 | -1.2400 | 0.5176 | 6.1406 | 0.1102 |
| 6 | -3.8812 | -4.3942 | 1.6374 | -1.9649 | 4.5013 | -1.9764 | 2.4705 | 5.6921 | 1.4418 |
| 8 | -4.3211 | -3.0902 | 1.9459 | -2.5350 | 3.2561 | -2.3076 | 2.9840 | 4.6500 | 2.2445 |
| 6 | -5.1148 | -2.7579 | -1.8782 | -3.7256 | 4.4619 | 1.0919 | 4.4963 | 3.9653 | -0.6246 |
| 6 | -4.7545 | -1.6744 | -2.9015 | -4.2777 | 3.9100 | 2.4090 | 5.2719 | 3.4428 | -1.8344 |
| 8 | -3.5476 | -2.0184 | -3.5717 | -3.4189 | 4.3071 | 3.4577 | 4.7687 | 4.0982 | -2.9813 |
| 6 | -4.5839 | -0.3167 | -2.2304 | -4.3755 | 2.3968 | 2.3139 | 5.1031 | 1.9371 | -1.9572 |

| | | | | | | | | | |
|---|---|---|---|---|---|---|---|---|---|
| 8 | -4.5322 | 0.6991 | -3.2053 | -4.9796 | 1.9231 | 3.4950 | 5.9369 | 1.5092 | -3.0047 |
| 6 | -5.6189 | -0.0163 | -1.1423 | -5.1756 | 2.0197 | 1.0769 | 5.4526 | 1.2732 | -0.6341 |
| 8 | -4.9828 | 0.9699 | -0.3380 | -5.1107 | 0.5980 | 1.0053 | 5.2025 | -0.1196 | -0.7761 |
| 6 | -5.9737 | -1.2445 | -0.2883 | -4.5684 | 2.6627 | -0.1678 | 4.5950 | 1.8679 | 0.4829 |
| 8 | -6.2269 | -2.3730 | -1.1250 | -4.5595 | 4.0804 | 0.0232 | 4.8931 | 3.2764 | 0.5426 |
| 6 | -7.2445 | -1.0378 | 0.5327 | -5.2828 | 2.3333 | -1.4642 | 4.8694 | 1.2533 | 1.8406 |
| 8 | -7.0209 | -0.1249 | 1.5881 | -6.6888 | 2.3275 | -1.3248 | 6.2574 | 1.0984 | 2.0165 |
| 6 | -6.7652 | -0.7642 | 2.8318 | -7.3124 | 2.2784 | -2.6030 | 6.6166 | 0.8826 | 3.3723 |
| 6 | -6.3129 | 0.3024 | 3.8089 | -8.6606 | 1.5999 | -2.4717 | 8.0026 | 0.2706 | 3.3687 |
| 8 | -5.0678 | 0.8575 | 3.4410 | -8.5119 | 0.2345 | -2.1247 | 7.9884 | -1.0120 | 2.7813 |
| 6 | -5.6694 | 2.1267 | 0.0631 | -6.3202 | -0.0980 | 0.8702 | 6.3042 | -0.9892 | -0.7129 |
| 6 | -5.6874 | 3.1996 | -1.0323 | -6.2790 | -1.3065 | 1.7948 | 6.1789 | -2.0184 | -1.8297 |
| 8 | -6.3336 | 2.6859 | -2.1875 | -6.0816 | -0.8314 | 3.1136 | 6.1597 | -1.3436 | -3.0718 |
| 6 | -4.2750 | 3.6425 | -1.3724 | -5.1482 | -2.2254 | 1.3704 | 4.9053 | -2.8222 | -1.6386 |
| 8 | -4.3753 | 4.7118 | -2.2838 | -5.2149 | -3.3834 | 2.1657 | 4.8884 | -3.8131 | -2.6370 |
| 6 | -3.5553 | 4.0524 | -0.1014 | -5.1913 | -2.5475 | -0.1248 | 4.7905 | -3.4118 | -0.2318 |
| 8 | -2.2311 | 4.3926 | -0.4835 | -3.8880 | -3.0297 | -0.4070 | 3.4222 | -3.8001 | -0.1352 |
| 6 | -3.5808 | 2.9107 | 0.9094 | -5.4305 | -1.2969 | -0.9764 | 5.1285 | -2.3519 | 0.8321 |
| 8 | -4.9733 | 2.6257 | 1.1816 | -6.5085 | -0.5298 | -0.4499 | 6.3439 | -1.6690 | 0.5148 |
| 6 | -2.8715 | 3.2532 | 2.2174 | -5.7746 | -1.5833 | -2.4323 | 5.3068 | -2.8686 | 2.2528 |
| 8 | -3.0421 | 4.6068 | 2.5980 | -7.0089 | -2.2466 | -2.5607 | 4.1753 | -3.5710 | 2.6965 |
| 6 | -1.7119 | 5.6038 | -0.0223 | -3.6928 | -4.1317 | -1.2533 | 3.1029 | -5.0275 | 0.4745 |
| 6 | -1.0480 | 6.3172 | -1.2008 | -3.2737 | -5.3229 | -0.3945 | 2.6233 | -6.0234 | -0.5872 |
| 8 | -2.0055 | 6.4453 | -2.2329 | -4.2984 | -5.5908 | 0.5436 | 3.6219 | -6.1392 | -1.5847 |
| 6 | 0.1690 | 5.5367 | -1.6673 | -1.9898 | -4.9685 | 0.3404 | 1.3090 | -5.5726 | -1.2057 |
| 8 | 0.7973 | 6.2953 | -2.6719 | -1.6343 | -6.0966 | 1.1168 | 0.8837 | -6.6051 | -2.0699 |
| 6 | 1.0865 | 5.2897 | -0.4793 | -0.9116 | -4.5244 | -0.6477 | 0.2910 | -5.2663 | -0.1189 |
| 8 | 2.1842 | 4.5278 | -0.9499 | 0.2135 | -4.0132 | 0.0651 | -0.8505 | -4.6735 | -0.7268 |
| 6 | 0.3187 | 4.5496 | 0.6159 | -1.4644 | -3.3904 | -1.5211 | 0.8923 | -4.2802 | 0.8750 |
| 8 | -0.7772 | 5.3877 | 1.0155 | -2.6698 | -3.8267 | -2.1605 | 2.0886 | -4.8319 | 1.4187 |
| 6 | 1.1572 | 4.2479 | 1.8352 | -0.5017 | -2.9474 | -2.6051 | -0.0332 | -3.9927 | 2.0321 |
| 8 | 1.7719 | 5.4283 | 2.2917 | -0.1565 | -4.0351 | -3.4429 | -0.2953 | -5.1915 | 2.7289 |
| 6 | 2.7990 | 5.1780 | 3.2348 | 1.2329 | -4.3076 | -3.4690 | -1.4934 | -5.1354 | 3.4750 |
| 6 | 2.3896 | 4.2997 | 4.4167 | 2.0128 | -3.2454 | -4.2435 | -1.5517 | -4.0014 | 4.4960 |
| 8 | 2.5091 | 2.9071 | 4.1542 | 3.3671 | -3.6234 | -4.3817 | -1.9362 | -2.7515 | 3.9336 |
| 6 | 3.4695 | 5.0476 | -0.7499 | 1.1042 | -4.9879 | 0.5710 | -1.9838 | -5.4878 | -0.8525 |
| 6 | 4.2559 | 4.8593 | -2.0538 | 1.3491 | -4.6386 | 2.0521 | -2.5602 | -5.2433 | -2.2564 |
| 8 | 3.5505 | 5.4951 | -3.1013 | 0.0977 | -4.4577 | 2.6993 | -1.5563 | -5.4782 | -3.2325 |
| 6 | 4.4116 | 3.3743 | -2.3188 | 2.2077 | -3.3844 | 2.1798 | -3.1142 | -3.8309 | -2.3373 |
| 8 | 5.1674 | 3.1930 | -3.4939 | 2.5952 | -3.2112 | 3.5181 | -3.7451 | -3.6367 | -3.5766 |
| 6 | 5.0677 | 2.7034 | -1.1228 | 3.4166 | -3.4735 | 1.2522 | -4.0810 | -3.5957 | -1.1832 |
| 8 | 5.0223 | 1.3173 | -1.4086 | 4.0908 | -2.2218 | 1.3435 | -4.4303 | -2.2243 | -1.2587 |
| 6 | 4.2645 | 2.9923 | 0.1472 | 2.9560 | -3.7517 | -0.1746 | -3.4255 | -3.8869 | 0.1655 |
| 8 | 4.1244 | 4.4056 | 0.3152 | 2.2964 | -5.0151 | -0.1683 | -2.9243 | -5.2259 | 0.1604 |
| 6 | 4.9102 | 2.4171 | 1.4118 | 4.0808 | -3.7879 | -1.2092 | -4.4058 | -3.7383 | 1.3441 |
| 8 | 3.9968 | 1.6852 | 2.2102 | 4.1723 | -2.5749 | -1.9429 | -4.1246 | -2.6296 | 2.1663 |

| | | | | | | | | | |
|---|---|---|---|---|---|---|---|---|---|
| 6 | 6.1895 | 0.5524 | -1.3714 | 5.4862 | -2.2009 | 1.4963 | -5.7683 | -1.8329 | -1.3001 |
| 6 | 6.0385 | -0.4986 | -2.4718 | 5.8233 | -1.2100 | 2.6077 | -5.8559 | -0.7033 | -2.3230 |
| 8 | 5.8695 | 0.1888 | -3.6961 | 5.1038 | -1.5894 | 3.7624 | -5.4462 | -1.2124 | -3.5755 |
| 6 | 4.8292 | -1.3676 | -2.1467 | 5.4448 | 0.1905 | 2.1655 | -4.9471 | 0.4409 | -1.8819 |
| 8 | 4.7145 | -2.3911 | -3.1101 | 5.8878 | 1.0780 | 3.1675 | -5.0541 | 1.4954 | -2.8141 |
| 6 | 4.9497 | -1.9302 | -0.7372 | 6.0236 | 0.5522 | 0.8001 | -5.2951 | 0.8632 | -0.4622 |
| 8 | 3.7192 | -2.5882 | -0.4785 | 5.4015 | 1.8052 | 0.5425 | -4.2623 | 1.7489 | -0.0310 |
| 6 | 5.1860 | -0.8021 | 0.2705 | 5.6984 | -0.5439 | -0.2284 | -5.3286 | -0.3451 | 0.4830 |
| 8 | 6.3542 | -0.0788 | -0.1207 | 6.1238 | -1.8159 | 0.3047 | -6.2008 | -1.3489 | -0.0436 |
| 6 | 5.3723 | -1.2885 | 1.6901 | 6.4125 | -0.3995 | -1.5711 | -5.8170 | 0.0226 | 1.8691 |
| 8 | 6.4055 | -2.2504 | 1.7159 | 7.7693 | -0.0284 | -1.4267 | -7.0512 | 0.7078 | 1.7522 |
| 6 | 3.7645 | -3.7244 | 0.3544 | 5.7048 | 2.5007 | -0.6321 | -4.6527 | 2.9180 | 0.6769 |
| 6 | 3.4756 | -4.9635 | -0.4930 | 5.6492 | 3.9912 | -0.3175 | -4.7289 | 4.0949 | -0.2983 |
| 8 | 4.3508 | -4.9449 | -1.6058 | 6.6016 | 4.3047 | 0.6676 | -5.6881 | 3.8095 | -1.3000 |
| 6 | 2.0293 | -4.9829 | -0.9642 | 4.2493 | 4.3487 | 0.1668 | -3.3612 | 4.3202 | -0.9247 |
| 8 | 1.8354 | -6.2190 | -1.6108 | 4.2507 | 5.7474 | 0.3855 | -3.4722 | 5.4244 | -1.7966 |
| 6 | 1.0702 | -4.7497 | 0.1996 | 3.1957 | 3.9034 | -0.8448 | -2.3291 | 4.5286 | 0.1746 |
| 8 | -0.2422 | -4.5975 | -0.3230 | 1.8884 | 4.0005 | -0.2802 | -1.0251 | 4.4834 | -0.3785 |
| 6 | 1.4821 | -3.4466 | 0.8860 | 3.4079 | 2.4342 | -1.2369 | -2.3822 | 3.3682 | 1.1616 |
| 8 | 2.8167 | -3.5949 | 1.3762 | 4.7596 | 2.2237 | -1.6454 | -3.7059 | 3.1815 | 1.6692 |
| 6 | 0.5894 | -3.0171 | 2.0227 | 2.5057 | 2.0333 | -2.3857 | -1.4153 | 3.5539 | 2.3186 |
| 8 | 0.5934 | -3.9957 | 3.0392 | 2.7198 | 2.9089 | -3.4679 | -1.4024 | 4.9060 | 2.7091 |
| 6 | 0.0834 | -3.5267 | 4.2714 | 1.7740 | 2.7252 | -4.5043 | -0.4631 | 5.1727 | 3.7347 |
| 6 | -1.4362 | -3.4863 | 4.3138 | 0.3576 | 3.1287 | -4.0762 | -0.4755 | 6.6746 | 3.9369 |
| 8 | -1.9911 | -2.5815 | 3.3658 | -0.5231 | 2.0390 | -3.8284 | -0.0418 | 7.3558 | 2.7761 |
| 1 | -0.5068 | -6.6327 | -0.0588 | 1.9878 | 6.0099 | -0.7457 | -1.2285 | 6.4916 | -0.8369 |
| 1 | -2.3948 | -6.7290 | -1.6869 | 0.5207 | 6.7048 | 1.0796 | 0.4513 | 6.3560 | -2.6582 |
| 1 | -1.1305 | -5.8480 | -3.5449 | 2.1016 | 4.6295 | 2.1361 | -1.0243 | 3.9590 | -2.6931 |
| 1 | -1.7726 | -3.7593 | -1.9708 | 0.0597 | 3.7279 | 1.6050 | 0.8426 | 3.4786 | -1.7091 |
| 1 | -3.3038 | -3.8691 | -3.6143 | -1.5804 | 4.7494 | 2.9957 | 2.8112 | 4.0005 | -3.1598 |
| 1 | -4.3579 | -5.0080 | -0.9553 | -1.7828 | 5.8512 | 0.4087 | 2.8165 | 5.7628 | -1.3208 |
| 1 | -2.1816 | -3.5318 | 0.6338 | -0.5225 | 3.3410 | -0.8512 | 1.0852 | 4.2293 | 0.7102 |
| 1 | -3.4093 | -4.8783 | 2.5084 | -1.5124 | 4.9939 | -2.8544 | 1.8737 | 6.3470 | 2.0907 |
| 1 | -4.7575 | -4.9992 | 1.3596 | -2.7859 | 5.1437 | -1.6233 | 3.2761 | 6.3102 | 1.0046 |
| 1 | -3.6435 | -2.7256 | 2.5389 | -1.9046 | 2.7803 | -2.8778 | 3.6643 | 4.1707 | 1.7500 |
| 1 | -5.3813 | -3.6983 | -2.3837 | -3.7152 | 5.5631 | 1.1021 | 4.7150 | 5.0309 | -0.4522 |
| 1 | -5.5494 | -1.6187 | -3.6574 | -5.2929 | 4.3201 | 2.5561 | 6.3429 | 3.6686 | -1.6883 |
| 1 | -2.8266 | -1.8231 | -2.9505 | -3.6568 | 3.7884 | 4.2371 | 5.1820 | 3.6762 | -3.7469 |
| 1 | -3.6074 | -0.3430 | -1.7026 | -3.3513 | 1.9831 | 2.1978 | 4.0362 | 1.7255 | -2.1773 |
| 1 | -5.1739 | 1.3874 | -2.9662 | -5.2119 | 0.9876 | 3.3702 | 5.8329 | 0.5484 | -3.1156 |
| 1 | -6.5384 | 0.3778 | -1.6098 | -6.2227 | 2.3476 | 1.1891 | 6.5131 | 1.4479 | -0.3890 |
| 1 | -5.1486 | -1.4803 | 0.4044 | -3.5245 | 2.3288 | -0.2871 | 3.5244 | 1.7393 | 0.2529 |
| 1 | -7.5783 | -2.0161 | 0.9161 | -4.9481 | 1.3342 | -1.8090 | 4.3506 | 0.2759 | 1.9139 |
| 1 | -8.0457 | -0.6366 | -0.1079 | -4.9519 | 3.0642 | -2.2192 | 4.4393 | 1.9121 | 2.6172 |
| 1 | -6.6880 | 1.8842 | 0.4022 | -7.1689 | 0.5596 | 1.1091 | 7.2447 | -0.4243 | -0.7949 |
| 1 | -6.2453 | 4.0671 | -0.6374 | -7.2403 | -1.8405 | 1.7067 | 7.0480 | -2.6962 | -1.7634 |

| | | | | | | | | | |
|---|---|---|---|---|---|---|---|---|---|
| 1 | -6.3254 | 3.3951 | -2.8451 | -6.0195 | -1.5997 | 3.6956 | 5.8639 | -1.9776 | -3.7381 |
| 1 | -3.7380 | 2.7813 | -1.8189 | -4.1950 | -1.6811 | 1.5479 | 4.0485 | -2.1284 | -1.7608 |
| 1 | -3.4962 | 5.1201 | -2.3689 | -4.7419 | -4.0978 | 1.7055 | 4.2597 | -4.5061 | -2.3700 |
| 1 | -4.0604 | 4.9264 | 0.3447 | -5.9640 | -3.3098 | -0.3298 | 5.4559 | -4.2901 | -0.1440 |
| 1 | -3.1096 | 2.0125 | 0.4757 | -4.4986 | -0.7063 | -0.9493 | 4.2905 | -1.6406 | 0.8537 |
| 1 | -1.8029 | 3.0001 | 2.1037 | -4.9795 | -2.2110 | -2.8652 | 5.5481 | -1.9996 | 2.8941 |
| 1 | -3.2662 | 2.5922 | 3.0039 | -5.7735 | -0.6263 | -2.9838 | 6.1716 | -3.5494 | 2.2956 |
| 1 | -2.2055 | 5.0510 | 2.4088 | -7.6960 | -1.5630 | -2.5490 | 3.3828 | -3.0796 | 2.4247 |
| 1 | -2.5075 | 6.2300 | 0.4107 | -4.6003 | -4.3527 | -1.8384 | 3.9697 | -5.4201 | 1.0247 |
| 1 | -0.7107 | 7.3110 | -0.8546 | -3.1008 | -6.1949 | -1.0490 | 2.4603 | -6.9962 | -0.0890 |
| 1 | -1.5262 | 6.7176 | -3.0265 | -3.9316 | -6.2443 | 1.1567 | 3.2431 | -6.6857 | -2.2871 |
| 1 | -0.1697 | 4.5552 | -2.0552 | -2.2178 | -4.1052 | 0.9955 | 1.4965 | -4.6343 | -1.7652 |
| 1 | 1.6383 | 5.8628 | -2.9015 | -1.1516 | -5.7776 | 1.8953 | 0.1053 | -6.2936 | -2.5611 |
| 1 | 1.4338 | 6.2527 | -0.0669 | -0.6029 | -5.3644 | -1.2965 | 0.0085 | -6.1872 | 0.4200 |
| 1 | -0.0745 | 3.5926 | 0.2242 | -1.6785 | -2.5191 | -0.8745 | 1.1134 | -3.3332 | 0.3517 |
| 1 | 1.9140 | 3.4824 | 1.5710 | 0.3864 | -2.5047 | -2.1216 | -0.9685 | -3.5555 | 1.6356 |
| 1 | 0.4948 | 3.8130 | 2.6043 | -0.9822 | -2.1543 | -3.1997 | 0.4514 | -3.2415 | 2.6809 |
| 1 | 3.4191 | 6.1131 | -0.4779 | 0.6662 | -5.9909 | 0.4800 | -1.7095 | -6.5491 | -0.7425 |
| 1 | 5.2546 | 5.3134 | -1.9226 | 1.8522 | -5.4798 | 2.5488 | -3.3729 | -5.9633 | -2.4375 |
| 1 | 3.9069 | 5.1495 | -3.9303 | -0.3088 | -3.6909 | 2.2656 | -1.1460 | -4.6302 | -3.4435 |
| 1 | 3.3988 | 2.9402 | -2.4301 | 1.5819 | -2.5350 | 1.8295 | -2.2688 | -3.1224 | -2.1997 |
| 1 | 5.2493 | 2.2354 | -3.6421 | 3.2815 | -2.5222 | 3.5447 | -4.1581 | -2.7557 | -3.5634 |
| 1 | 6.1119 | 3.0499 | -1.0137 | 4.0842 | -4.2869 | 1.5882 | -4.9743 | -4.2335 | -1.3160 |
| 1 | 3.2664 | 2.5307 | 0.0168 | 2.2292 | -2.9759 | -0.4806 | -2.5965 | -3.1645 | 0.3052 |
| 1 | 5.3677 | 3.2456 | 1.9754 | 3.8755 | -4.6206 | -1.9028 | -4.4017 | -4.6868 | 1.9084 |
| 1 | 5.7173 | 1.7264 | 1.1268 | 5.0549 | -3.9779 | -0.7308 | -5.4281 | -3.5890 | 0.9649 |
| 1 | 7.0827 | 1.1771 | -1.5281 | 5.8572 | -3.2086 | 1.7377 | -6.4290 | -2.6748 | -1.5578 |
| 1 | 6.9462 | -1.1272 | -2.4888 | 6.9136 | -1.2428 | 2.7869 | -6.8992 | -0.3436 | -2.3631 |
| 1 | 5.6151 | -0.4612 | -4.3629 | 5.1959 | -0.8738 | 4.4049 | -5.3352 | -0.4570 | -4.1677 |
| 1 | 3.9412 | -0.7078 | -2.1744 | 4.3411 | 0.2385 | 2.0745 | -3.9030 | 0.0680 | -1.8827 |
| 1 | 4.3728 | -3.1863 | -2.6659 | 5.6866 | 1.9718 | 2.8610 | -5.3120 | 2.3084 | -2.3461 |
| 1 | 5.7883 | -2.6484 | -0.6980 | 7.1192 | 0.6635 | 0.8680 | -6.2738 | 1.3728 | -0.4475 |
| 1 | 4.3061 | -0.1321 | 0.2617 | 4.6085 | -0.5662 | -0.4159 | -4.3193 | -0.7785 | 0.5739 |
| 1 | 4.4203 | -1.7302 | 2.0403 | 5.9108 | 0.3764 | -2.1617 | -5.0614 | 0.6699 | 2.3555 |
| 1 | 5.6078 | -0.4300 | 2.3444 | 6.3036 | -1.3558 | -2.1052 | -5.9105 | -0.9013 | 2.4582 |
| 1 | 4.7509 | -3.7986 | 0.8404 | 6.6905 | 2.2017 | -1.0249 | -5.6174 | 2.7440 | 1.1811 |
| 1 | 3.6508 | -5.8591 | 0.1299 | 5.8451 | 4.5363 | -1.2605 | -5.0242 | 4.9933 | 0.2717 |
| 1 | 4.0604 | -5.6605 | -2.1881 | 6.3652 | 5.1877 | 0.9828 | -5.6182 | 4.5259 | -1.9478 |
| 1 | 1.8832 | -4.1370 | -1.6693 | 4.0718 | 3.8035 | 1.1132 | -3.0848 | 3.3969 | -1.4765 |
| 1 | 1.0305 | -6.1592 | -2.1516 | 3.5671 | 5.9413 | 1.0465 | -2.7307 | 5.3893 | -2.4223 |
| 1 | 1.1111 | -5.5795 | 0.9267 | 3.2644 | 4.5277 | -1.7525 | -2.5040 | 5.4860 | 0.6924 |
| 1 | 1.4385 | -2.6348 | 0.1371 | 3.1752 | 1.7924 | -0.3666 | -2.0651 | 2.4741 | 0.6045 |
| 1 | -0.4229 | -2.8461 | 1.6240 | 1.4626 | 2.0925 | -2.0111 | -0.4064 | 3.2403 | 1.9956 |
| 1 | 0.9628 | -2.0507 | 2.4146 | 2.7011 | 0.9887 | -2.6783 | -1.7213 | 2.8983 | 3.1537 |
| 1 | 3.6615 | 4.7072 | 2.7264 | 1.6240 | -4.4052 | -2.4379 | -2.3569 | -5.0442 | 2.7861 |
| 1 | 3.1136 | 6.1644 | 3.6013 | 1.3605 | -5.2797 | -3.9660 | -1.5734 | -6.0995 | 3.9954 |

| | | | | | | | | | |
|---|---|---|---|---|---|---|---|---|---|
| 1 | 1.3681 | 4.5572 | 4.7407 | 1.9189 | -2.2653 | -3.7398 | -0.5818 | -3.9187 | 5.0127 |
| 1 | 1.6921 | 2.5276 | 3.7814 | 3.8464 | -3.2020 | -3.6510 | -1.1792 | -2.3043 | 3.5204 |
| 1 | -7.6822 | -1.2613 | 3.1961 | -7.4130 | 3.2961 | -3.0174 | 6.5890 | 1.8334 | 3.9326 |
| 1 | -5.9790 | -1.5330 | 2.7144 | -6.6916 | 1.6869 | -3.3010 | 5.9066 | 0.1809 | 3.8479 |
| 1 | -6.1934 | -0.1474 | 4.8041 | -9.1907 | 1.6481 | -3.4318 | 8.3649 | 0.1651 | 4.4005 |
| 1 | -5.1798 | 1.3524 | 2.6130 | -7.9027 | 0.1990 | -1.3715 | 7.4708 | -0.9583 | 1.9630 |
| 1 | 0.4393 | -4.2170 | 5.0492 | 2.1170 | 3.3567 | -5.3334 | -0.7379 | 4.6451 | 4.6650 |
| 1 | 0.4963 | -2.5245 | 4.4958 | 1.7673 | 1.6756 | -4.8535 | 0.5436 | 4.8267 | 3.4244 |
| 1 | -1.7712 | -3.2469 | 5.3362 | -0.1239 | 3.7143 | -4.8706 | 0.1962 | 6.9478 | 4.7621 |
| 1 | -1.7527 | -1.6697 | 3.5844 | -0.1825 | 1.4283 | -3.1511 | -0.3312 | 6.8483 | 2.0032 |
| 1 | 3.6276 | 2.2438 | 2.9207 | 3.4423 | -1.9739 | -1.7071 | -3.3515 | -2.8162 | 2.7287 |
| 6 | 6.5790 | -2.8311 | 2.9974 | 8.7124 | -1.0888 | -1.3933 | -8.0256 | 0.3702 | 2.7313 |
| 6 | 7.8334 | -3.6783 | 2.9018 | 9.5803 | -0.9793 | -0.1524 | -8.9136 | -0.7556 | 2.2221 |
| 8 | 8.9319 | -2.8780 | 2.5130 | 8.8152 | -1.0731 | 1.0355 | -8.1796 | -1.9373 | 1.9690 |
| 1 | 6.7106 | -2.0376 | 3.7546 | 8.1916 | -2.0622 | -1.3929 | -7.5363 | 0.0777 | 3.6749 |
| 1 | 5.6962 | -3.4359 | 3.2739 | 9.3458 | -1.0492 | -2.2947 | -8.6268 | 1.2705 | 2.9229 |
| 1 | 7.6617 | -4.5008 | 2.1842 | 10.0921 | -0.0059 | -0.1407 | -9.4415 | -0.4123 | 1.3151 |
| 1 | 8.6216 | -2.3620 | 1.7577 | 8.0635 | -1.6498 | 0.8445 | -7.6338 | -1.7840 | 1.1841 |
| 1 | 3.0648 | 4.5190 | 5.2558 | 1.5647 | -3.1431 | -5.2445 | -2.3114 | -4.2485 | 5.2501 |
| 1 | 8.0670 | -4.1240 | 3.8775 | 10.3563 | -1.7643 | -0.1957 | -9.6752 | -0.9922 | 2.9792 |
| 1 | -1.8269 | -4.4791 | 4.0532 | 0.4607 | 3.7886 | -3.1932 | -1.4988 | 6.9820 | 4.2179 |
| 1 | -7.0943 | 1.0791 | 3.8842 | -9.2713 | 2.1305 | -1.7208 | 8.6922 | 0.9539 | 2.8395 |
| 6 | -2.9389 | -0.4069 | 1.6226 | -1.9563 | 0.3353 | -1.3144 | -1.8409 | -0.4893 | 1.8416 |
| 7 | -2.0102 | 0.2655 | 2.2600 | -0.6885 | 0.2083 | -1.6263 | -0.6332 | -0.9319 | 2.1078 |
| 6 | -0.8827 | 0.1670 | 1.4827 | -0.0593 | -0.1757 | -0.4658 | 0.1431 | -0.5766 | 1.0308 |
| 6 | -1.1570 | -0.5929 | 0.3498 | -0.9859 | -0.2866 | 0.5602 | -0.6388 | 0.1066 | 0.1073 |
| 7 | -2.4719 | -0.9523 | 0.4669 | -2.1886 | 0.0388 | -0.0038 | -1.8951 | 0.1368 | 0.6396 |
| 6 | 0.3855 | 0.7967 | 1.9244 | 1.3822 | -0.4734 | -0.4027 | 1.5952 | -0.8947 | 0.9629 |
| 8 | 0.4151 | 1.4603 | 2.9700 | 2.0243 | -0.8164 | -1.4086 | 2.1765 | -1.4603 | 1.8936 |
| 7 | 1.4647 | 0.5927 | 1.1503 | 1.9469 | -0.3495 | 0.8097 | 2.2009 | -0.5184 | -0.1812 |
| 7 | -0.2751 | -0.9109 | -0.6685 | -0.7702 | -0.6472 | 1.8785 | -0.2634 | 0.6458 | -1.1160 |
| 7 | -0.7618 | -1.5496 | -1.6510 | -1.8512 | -0.6084 | 2.5558 | -1.2153 | 1.1974 | -1.7357 |
| 7 | 0.1192 | -1.8645 | -2.5705 | -1.7307 | -0.9132 | 3.8031 | -0.8819 | 1.7388 | -2.8941 |
| 6 | -0.3999 | -2.3095 | -3.8547 | -2.9320 | -0.8406 | 4.6122 | -1.9905 | 1.9185 | -3.8271 |
| 6 | 1.4679 | -1.3319 | -2.4994 | -0.4415 | -1.2287 | 4.3882 | 0.4269 | 1.4714 | -3.4745 |
| 1 | -3.9717 | -0.4958 | 1.9476 | -2.7445 | 0.6571 | -1.9892 | -2.7150 | -0.6036 | 2.4765 |
| 1 | -2.9818 | -1.5974 | -0.1395 | -3.0808 | 0.0774 | 0.4896 | -2.6999 | 0.6255 | 0.2437 |
| 1 | 1.3837 | 0.0422 | 0.3028 | 1.3593 | -0.1814 | 1.6190 | 1.6741 | -0.0395 | -0.9033 |
| 1 | 2.3704 | 0.9466 | 1.4603 | 2.8592 | -0.7681 | 0.9586 | 3.2007 | -0.6234 | -0.3034 |
| 1 | -0.8097 | -1.4719 | -4.4384 | -3.0814 | -1.7862 | 5.1472 | -1.8308 | 2.8330 | -4.4121 |
| 1 | 0.4188 | -2.7670 | -4.4163 | -2.8585 | -0.0212 | 5.3394 | -2.0492 | 1.0636 | -4.5175 |
| 1 | -1.1736 | -3.0721 | -3.7172 | -3.7765 | -0.6625 | 3.9382 | -2.9328 | 1.9800 | -3.2700 |
| 1 | 2.0511 | -1.7387 | -3.3317 | -0.5879 | -1.4080 | 5.4560 | 0.5365 | 2.1046 | -4.3593 |
| 1 | 1.4508 | -0.2307 | -2.5499 | -0.0010 | -2.1219 | 3.9196 | 1.2225 | 1.7172 | -2.7633 |
| 1 | 1.9485 | -1.6261 | -1.5585 | 0.2516 | -0.3893 | 4.2386 | 0.5115 | 0.4076 | -3.7473 |

**Table S3.** Densities, viscosities and relative permittivities of pure water at temperatures $T$ = (288.15 to 318.15)K.

| T/K | $\rho_0$ /kg·m$^{-3}$ [a] | $10^3 \cdot \eta$ /Pa·s [a] | $\varepsilon_r$ [b] |
|---|---|---|---|
| 283.15 | 999.700 | 1.130 | 83.95 |
| 288.15 | 999.100 | 1.138 | 82.07 |
| 293.15 | 998.205 | 1.002 | 80.21 |
| 298.15 | 997.047 | 0.890 | 78.40 |
| 303.15 | 995.651 | 0.797 | 76.62 |
| 308.15 | 994.038 | 0.719 | 74.89 |
| 313.15 | 992.224 | 0.653 | 73.19 |
| 318.15 | 990.223 | 0.596 | 71.53 |

[a] values for water recommended by the International Association for the Properties of Water and Steam (IAPWS) calculated on the online property calculator (https://web1.hszg.de/thermo_fpc/)

[b]values for water calculated according to the IAPWS recommendations (http://www.iapws.org/relguide/dielec.pdf)

**Table S4**. The value of molar concentration of salt $C_{salt}$ [mol/dm$^3$], concentration of CD $C_{Cd}$ [mol/dm$^3$], molar conductivity $\Lambda$ [S·cm$^2$·mol$^{-1}$] for $\alpha$-cyclodextrin ($\alpha$-CD) with dacarbazine (DTIC) in water at all tested temperatures at pressure p = 0.1 MPa.[a]

| | | | $\alpha$-cyclodextrin ($\alpha$-CD) | | | | |
|---|---|---|---|---|---|---|---|
| T [K] | | | 283.15 | 298.15 | 303.15 | 308.15 | 313.15 |
| Nr | $c_{salt}$ [mol/dm$^3$] | $C_{Cd}$ [mol/dm$^3$] | $\Lambda_m$ [S·cm$^2$·mol$^{-1}$] | $\Lambda_m$ [S·cm$^2$·mol$^{-1}$] | $\Lambda_m$ [S·cm$^2$·mol$^{-1}$] | $\Lambda_m$ [S·cm$^2$·mol$^{-1}$] | $\Lambda_m$ [S·cm$^2$·mol$^{-1}$] |
| 1. | 0.00336 | 0.00000 | 58.6371 | 65.4451 | 70.4551 | 79.7197 | 85.7585 |
| 2. | 0.00332 | 0.00024 | 58.0358 | 64.3139 | 69.3139 | 78.3494 | 84.9254 |
| 3. | 0.00330 | 0.00034 | 57.7261 | 63.0466 | 68.0466 | 77.2996 | 84.3408 |
| 4. | 0.00327 | 0.00048 | 57.3521 | 61.8202 | 66.8202 | 76.3779 | 83.6545 |
| 5. | 0.00325 | 0.00061 | 56.9879 | 60.5763 | 65.5763 | 75.3714 | 82.8807 |
| 6. | 0.00321 | 0.00078 | 56.5004 | 59.3339 | 64.3339 | 73.8916 | 82.1244 |
| 7. | 0.00318 | 0.00092 | 56.1098 | 58.1578 | 63.1588 | 72.3621 | 81.3432 |
| 8. | 0.00315 | 0.00107 | 55.6821 | 57.0393 | 62.0393 | 70.9685 | 80.6511 |
| 9. | 0.00312 | 0.00122 | 55.1642 | 56.1847 | 61.1847 | 69.7074 | 79.9165 |
| 10 | 0.00308 | 0.00143 | 54.6724 | 55.6365 | 60.6365 | 68.7348 | 78.9239 |
| 11 | 0.00304 | 0.00163 | 54.0607 | 55.3173 | 60.3173 | 68.0572 | 77.8810 |
| 12 | 0.00300 | 0.00184 | 53.4228 | 55.1134 | 60.1134 | 67.6576 | 76.9524 |
| 13 | 0.00297 | 0.00201 | 52.9162 | 54.9841 | 59.9841 | 67.4632 | 75.9657 |
| 14 | 0.00293 | 0.00223 | 52.3630 | 54.8750 | 59.8750 | 67.2821 | 74.9964 |
| 15 | 0.00287 | 0.00251 | 51.7675 | 54.7947 | 59.7947 | 67.1296 | 74.0091 |
| 16 | 0.00281 | 0.00280 | 51.3344 | 54.7369 | 59.7369 | 67.0207 | 73.1524 |
| 17 | 0.00275 | 0.00311 | 51.0885 | 54.6984 | 59.6984 | 66.9516 | 72.6355 |
| 18 | 0.00269 | 0.00341 | 50.9508 | 54.6264 | 59.6264 | 66.8760 | 72.2723 |
| 19 | 0.00264 | 0.00368 | 50.8979 | 54.5517 | 59.5517 | 66.8308 | 72.1069 |
| 20 | 0.00258 | 0.00398 | 50.8093 | 54.5414 | 59.5414 | 66.7371 | 71.9432 |
| 21 | 0.00251 | 0.00431 | 50.8033 | 54.4920 | 59.4920 | 66.7140 | 71.7653 |
| 22 | 0.00245 | 0.00462 | 50.7557 | 54.4535 | 59.4635 | 66.6466 | 71.6055 |
| 23 | 0.00239 | 0.00491 | 50.7283 | 54.4237 | 59.4237 | 66.6352 | 71.4917 |
| 24 | 0.00233 | 0.00522 | 50.6857 | 54.3892 | 59.3892 | 66.5354 | 71.2195 |
| 25 | 0.00227 | 0.00550 | 50.6484 | 54.3223 | 59.3623 | 66.4586 | 71.1472 |
| 26 | 0.00221 | 0.00578 | 50.6012 | 54.3123 | 59.3023 | 66.3054 | 70.9917 |
| 27 | 0.00217 | 0.00608 | 50.5936 | 54.2541 | 59.2533 | 66.2723 | 70.9087 |
| 28 | 0.00210 | 0.00638 | 50.5815 | 54.2024 | 59.2124 | 66.1932 | 70.7597 |

[a] Standard uncertainties are $u(T)$ = 0.01 K, $u(p)$ = 0.05 MPa, $u(c) = 10^{-4} \cdot c$, and the combined expanded uncertainty is $U_c(\Lambda) = 0.0005 \cdot \Lambda$ (level of confidence = 0.95).

**Table S5**. The value of molar concentration of salt $C_{salt}$ [mol/dm³], concentration of CD $C_{Cd}$ [mol/dm³], molar conductivity Λ [S·cm²·mol⁻¹] for hydroxypropyl-β-cyclodextrin (HP-β-CD) with dacarbazine (DTIC) in water at all tested temperatures at pressure p = 0.1 MPa.[a]

| hydroxypropyl-β-cyclodextrin (HP-β-CD) | | | | | | | |
|---|---|---|---|---|---|---|---|
| T [K] | | | 283.15 | 298.15 | 303.15 | 308.15 | 313.15 |
| Nr | $c_{salt}$ mol/dm³] | $C_{Cd}$ [mol/dm³] | $\Lambda_m$ [S·cm²·mol⁻¹] | $\Lambda_m$ [S·cm²·mol⁻¹] | $\Lambda_m$ [S·cm²·mol⁻¹] | $\Lambda_m$ S·cm²·mol⁻¹] | $\Lambda_m$ S·cm²·mol⁻¹] |
| 1. | 0.00304 | 0.00000 | 64.7252 | 67.5068 | 72.7309 | 82.0947 | 88.0679 |
| 2. | 0.00301 | 0.00026 | 63.9428 | 66.3555 | 71.5796 | 81.1891 | 86.6698 |
| 3. | 0.00299 | 0.00039 | 63.4142 | 65.7218 | 70.9460 | 80.5376 | 85.9741 |
| 4. | 0.00297 | 0.00058 | 62.8972 | 64.8200 | 70.0442 | 79.6505 | 85.2783 |
| 5. | 0.00296 | 0.00067 | 62.3811 | 64.2355 | 69.4596 | 79.2274 | 84.3041 |
| 6. | 0.00294 | 0.00080 | 61.8538 | 63.3726 | 68.5967 | 78.6690 | 83.4709 |
| 7. | 0.00292 | 0.00094 | 61.2259 | 62.8278 | 68.0519 | 78.0361 | 82.5384 |
| 8. | 0.00290 | 0.00108 | 60.6871 | 62.0017 | 67.2258 | 77.3293 | 81.9537 |
| 9. | 0.00289 | 0.00119 | 60.1615 | 61.2968 | 66.5209 | 76.7920 | 80.9923 |
| 10 | 0.00285 | 0.00147 | 59.3895 | 60.2829 | 65.5070 | 75.3943 | 79.8545 |
| 11 | 0.00283 | 0.00169 | 58.6626 | 59.3302 | 64.5544 | 74.3259 | 78.8135 |
| 12 | 0.00280 | 0.00190 | 57.9480 | 58.4615 | 63.6856 | 73.3497 | 77.5867 |
| 13 | 0.00277 | 0.00210 | 57.2476 | 57.6813 | 62.9054 | 72.4974 | 76.4282 |
| 14 | 0.00274 | 0.00237 | 56.5913 | 57.0319 | 62.2560 | 71.3975 | 75.5683 |
| 15 | 0.00269 | 0.00273 | 55.8084 | 56.2858 | 61.5099 | 70.2155 | 74.2275 |
| 16 | 0.00266 | 0.00299 | 55.1194 | 55.7450 | 60.9691 | 69.5657 | 73.0799 |
| 17 | 0.00261 | 0.00335 | 54.6044 | 55.3292 | 60.5533 | 68.9600 | 72.2722 |
| 18 | 0.00258 | 0.00361 | 54.2090 | 55.0443 | 60.2685 | 68.6863 | 71.7202 |
| 19 | 0.00254 | 0.00393 | 53.8408 | 54.8304 | 60.0545 | 68.4101 | 71.1600 |
| 20 | 0.00250 | 0.00423 | 53.5442 | 54.6413 | 59.8654 | 68.2121 | 70.9371 |
| 21 | 0.00246 | 0.00454 | 53.4012 | 54.4890 | 59.7131 | 68.0479 | 70.6896 |
| 22 | 0.00242 | 0.00482 | 53.1831 | 54.3471 | 59.5712 | 67.9302 | 70.4649 |
| 23 | 0.00238 | 0.00512 | 52.9968 | 54.2181 | 59.4422 | 67.8199 | 70.2833 |
| 24 | 0.00235 | 0.00539 | 52.7911 | 54.1024 | 59.3265 | 67.7111 | 70.0309 |
| 25 | 0.00231 | 0.00570 | 52.6260 | 53.9617 | 59.1859 | 67.6226 | 69.8413 |
| 26 | 0.00226 | 0.00608 | 52.3958 | 53.9405 | 59.1647 | 67.5317 | 69.5544 |
| 27 | 0.00184 | 0.00629 | 52.3047 | 53.8476 | 59.0718 | 67.5663 | 69.5246 |
| 28 | 0.00178 | 0.00642 | 52.2985 | 53.8112 | 58.9981 | 67.5562 | 69.4824 |

**Table S6**. The value of molar concentration of salt $C_{salt}$ [mol/dm³], concentration of CD $C_{Cd}$ [mol/dm³], molar conductivity Λ [S·cm²·mol⁻¹] for hydroxyethyl-β-cyclodextrin (HE-β-CD) with dacarbazine (DTIC) in water at all tested temperatures at pressure p = 0.1 MPa.[a]

| hydroxyethyl-β-cyclodextrin (HE-β-CD) | | | | | | | |
|---|---|---|---|---|---|---|---|
| T [K] | | | 283.15 | 298.15 | 303.15 | 308.15 | 313.15 |
| Nr | $c_{salt}$ [mol/dm³] | $C_{Cd}$ [mol/dm³] | $\Lambda_m$ [S·cm²·mol⁻¹] | $\Lambda_m$ [S·cm²·mol⁻¹] | $\Lambda_m$ [S·cm²·mol⁻¹] | $\Lambda_m$ S·cm²·mol⁻¹] | $\Lambda_m$ S·cm²·mol⁻¹] |
| 1. | 0.00296 | 0.00000 | 60.9992 | 67.7315 | 72.8980 | 82.0997 | 87.9826 |
| 2. | 0.00292 | 0.00026 | 60.4878 | 67.2201 | 72.3865 | 81.5882 | 87.4711 |
| 3. | 0.00290 | 0.00033 | 60.3117 | 67.0440 | 72.2104 | 81.4122 | 87.2950 |
| 4. | 0.00287 | 0.00047 | 59.9661 | 66.6984 | 71.8648 | 81.0665 | 86.9494 |
| 5. | 0.00285 | 0.00062 | 59.6837 | 66.4160 | 71.5824 | 80.7842 | 86.6670 |
| 6. | 0.00282 | 0.00076 | 59.2792 | 66.0115 | 71.1779 | 80.3796 | 86.2625 |
| 7. | 0.00279 | 0.00091 | 59.0077 | 65.7400 | 70.9064 | 80.1081 | 85.9910 |

| | | | | | | | |
|---|---|---|---|---|---|---|---|
| 8. | 0.00276 | 0.00108 | 58.6647 | 65.3970 | 70.5634 | 79.7651 | 85.6480 |
| 9. | 0.00274 | 0.00121 | 58.3361 | 65.0684 | 70.2348 | 79.4366 | 85.3194 |
| 10 | 0.00270 | 0.00142 | 57.8199 | 64.5522 | 69.7186 | 78.9203 | 84.8032 |
| 11 | 0.00266 | 0.00163 | 57.4755 | 64.2078 | 69.3742 | 78.5759 | 84.4588 |
| 12 | 0.00262 | 0.00183 | 57.0926 | 63.8249 | 68.9913 | 78.1930 | 84.0759 |
| 13 | 0.00258 | 0.00204 | 56.6983 | 63.4306 | 68.5970 | 77.7988 | 83.6816 |
| 14 | 0.00257 | 0.00221 | 56.3731 | 63.1054 | 68.2718 | 77.4736 | 83.3564 |
| 15 | 0.00249 | 0.00251 | 56.0833 | 62.8156 | 67.9820 | 77.1837 | 83.0666 |
| 16 | 0.00244 | 0.00280 | 55.8699 | 62.6022 | 67.7686 | 76.9704 | 82.8532 |
| 17 | 0.00238 | 0.00309 | 55.7407 | 62.4730 | 67.6394 | 76.8411 | 82.7240 |
| 18 | 0.00233 | 0.00339 | 55.6786 | 62.4109 | 67.5773 | 76.7790 | 82.6619 |
| 19 | 0.00227 | 0.00372 | 55.7370 | 62.4693 | 67.6357 | 76.8374 | 82.7203 |
| 20 | 0.00222 | 0.00398 | 55.7213 | 62.4536 | 67.6200 | 76.8218 | 82.7046 |
| 21 | 0.00216 | 0.00428 | 55.6937 | 62.4260 | 67.5924 | 76.7941 | 82.6770 |
| 22 | 0.00211 | 0.00457 | 55.7175 | 62.4498 | 67.6162 | 76.8179 | 82.7008 |
| 23 | 0.00206 | 0.00484 | 55.6751 | 62.4074 | 67.5738 | 76.7755 | 82.6584 |
| 24 | 0.00199 | 0.00518 | 55.7128 | 62.4451 | 67.6115 | 76.8132 | 82.6961 |
| 25 | 0.00194 | 0.00546 | 55.7138 | 62.4461 | 67.6125 | 76.8142 | 82.6971 |
| 26 | 0.00188 | 0.00576 | 55.7197 | 62.4520 | 67.6184 | 76.8201 | 82.7030 |
| 27 | 0.00183 | 0.00604 | 55.6691 | 62.4014 | 67.5678 | 76.7695 | 82.6524 |
| 28 | 0.00179 | 0.00625 | 55.6433 | 62.3756 | 67.5620 | 76.7537 | 82.6266 |

**Table 7.** ΔS [kJ/mol·K] in the temperature range 293.15–313.15 K calculated for the α-CD:DTIC, HP-β-CD:DTIC and HE-β-CD:DTIC complexes presented in Figure 4 of the main article. Experimental values (EXP) are written in blue.

| **α-CD:DTIC** | **ΔS PCM** | **ΔS CPCM** | **ΔS SMD** | **ΔS Onsag** | **ΔS EXP** |
|---|---|---|---|---|---|
| | | | **293.15** | | |
| **K02_03** | **-0.25** | **-0.25** | **-0.26** | **-0.25** | |
| **K01_01** | -0.26 | -0.26 | -0.26 | -0.26 | **-0.2506** |
| **K03_03** | -0.26 | -0.26 | -0.25 | -0.25 | |
| | | | **298.15** | | |
| **K02_03** | -0.25 | -0.25 | -0.26 | -0.25 | |
| **K01_01** | -0.26 | -0.26 | -0.26 | -0.26 | **-0.2502** |
| **K03_03** | -0.26 | -0.25 | -0.25 | -0.25 | |
| | | | **303.15** | | |
| **K02_03** | -0.25 | -0.25 | -0.26 | -0.25 | |
| **K01_01** | -0.26 | -0.22 | -0.26 | -0.26 | **-0.2594** |
| **K03_03** | -0.26 | -0.25 | -0.25 | -0.25 | |
| | | | **308.15** | | |
| **K02_03** | -0.25 | -0.25 | -0.26 | -0.25 | |
| **K01_01** | -0.26 | -0.26 | -0.26 | -0.27 | **-0.3123** |
| **K03_03** | -0.26 | -0.25 | -0.25 | -0.25 | |
| | | | **313.15** | | |
| **K02_03** | -0.25 | -0.25 | -0.26 | -0.25 | |
| **K01_01** | -0.26 | -0.27 | -0.26 | -0.26 | **-0.3417** |
| **K03_03** | **-0.25** | **-0.25** | **-0.25** | **-0.25** | |
| **HP-β-CD:DTIC** | **ΔS PCM** | **ΔS CPCM** | **ΔS SMD** | **ΔS Onsag** | **ΔS EXP** |
| | | | **293.15** | | |

| | | | | | |
|---|---|---|---|---|---|
| **K03_03** | -0.27 | -0.27 | -0.29 | -0.26 | **-0.29** |
| **K04_03** | -0.26 | -0.26 | -0.28 | -0.26 | |
| **K04_01** | -0.27 | -0.27 | -0.30 | -0.29 | |
| **298.15** | | | | | |
| **K03_03** | -0.27 | -0.27 | -0.29 | -0.26 | **-0.31** |
| **K04_03** | -0.26 | -0.26 | -0.28 | -0.26 | |
| **K04_01** | -0.27 | -0.27 | -0.30 | -0.29 | |
| **303.15** | | | | | |
| **K03_03** | -0.27 | -0.27 | -0.29 | -0.25 | **-0.33** |
| **K04_03** | -0.26 | -0.26 | -0.28 | -0.25 | |
| **K04_01** | -0.27 | -0.27 | -0.30 | -0.27 | |
| **308.15** | | | | | |
| **K03_03** | -0.27 | -0.27 | -0.29 | -0.26 | **-0.35** |
| **K04_03** | -0.26 | -0.26 | -0.28 | -0.26 | |
| **K04_01** | -0.27 | -0.27 | -0.28 | -0.26 | |
| **313.15** | | | | | |
| **K03_03** | -0.27 | -0.27 | -0.29 | -0.26 | **-0.37** |
| **K04_03** | -0.26 | -0.26 | -0.28 | -0.26 | |
| **K04_01** | **-0.27** | **-0.27** | **-0.30** | **-0.29** | |
| **HE-β-CD:DTIC** | **ΔS PCM** | **ΔS CPCM** | **ΔS SMD** | **ΔS Onsag** | **ΔS EXP** |
| **293.15** | | | | | |
| **K04_01** | -0.32 | -0.32 | -0.33 | -0.32 | **-0.48** |
| **K03_02** | -0.30 | -0.30 | -0.32 | -0.31 | |
| **K03_03** | -0.30 | -0.30 | -0.32 | -0.30 | |
| **298.15** | | | | | |
| **K04_01** | -0.32 | -0.32 | -0.33 | -0.32 | **-0.46** |
| **K03_02** | -0.30 | -0.30 | -0.32 | -0.31 | |
| **K03_03** | -0.30 | -0.30 | -0.32 | -0.30 | |
| **303.15** | | | | | |
| **K04_01** | -0.32 | -0.32 | -0.33 | -0.32 | **-0.40** |
| **K03_02** | -0.30 | -0.30 | -0.32 | -0.31 | |
| **K03_03** | -0.30 | -0.30 | -0.32 | -0.30 | |
| **308.15** | | | | | |
| **K04_01** | -0.32 | -0.32 | -0.33 | -0.31 | **-0.38** |
| **K03_02** | -0.30 | -0.30 | -0.32 | -0.31 | |
| **K03_03** | -0.30 | -0.30 | -0.32 | -0.30 | |
| **313.15** | | | | | |
| **K04_01** | -0.32 | -0.32 | -0.33 | -0.30 | **-0.35** |
| **K03_02** | -0.30 | -0.30 | -0.32 | -0.31 | |
| **K03_03** | -0.30 | -0.30 | -0.32 | -0.30 | |

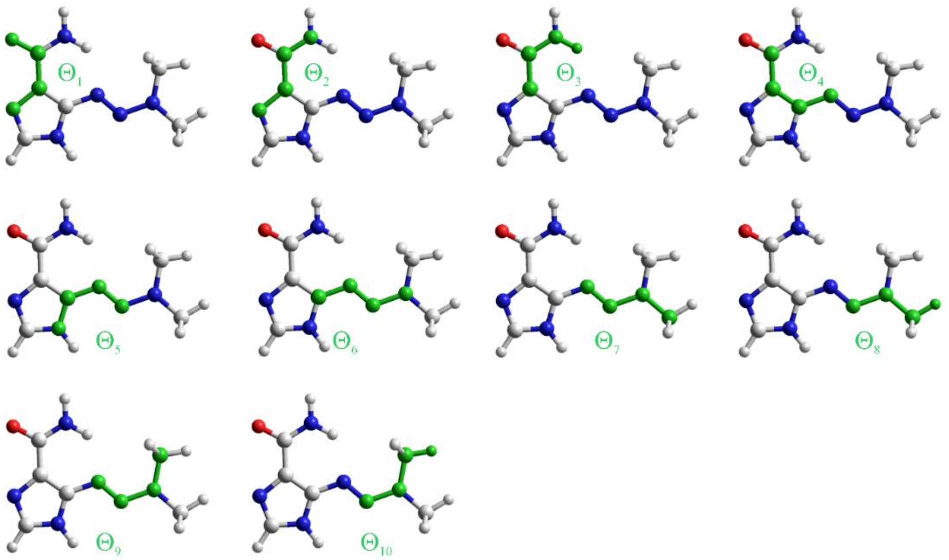


**Figure S6.** Torsion angles (θ; green highlight) marked in dacarbazine and changed during the conformational procedure performed in HyperChem.
